# Vitamin D opposes multilineage cell differentiation induced by Notch inhibition and BMP4 pathway activation in human colon organoids

**DOI:** 10.1038/s41419-024-06680-z

**Published:** 2024-04-29

**Authors:** Pilar Bustamante-Madrid, Antonio Barbáchano, David Albandea-Rodríguez, Javier Rodríguez-Cobos, Nuria Rodríguez-Salas, Isabel Prieto, Aurora Burgos, Jaime Martínez de Villarreal, Francisco X. Real, José Manuel González-Sancho, María Jesús Larriba, Miguel Lafarga, Alberto Muñoz, Asunción Fernández-Barral

**Affiliations:** 1 Instituto de Investigaciones Biomédicas Sols-Morreale, CSIC-UAM, 28029 Madrid, Spain; 2 Centro de Investigación Biomédica en Red-Cáncer (CIBERONC), 28029 Madrid, Spain; 3 Instituto de Investigación Sanitaria Hospital Universitario La Paz (IdiPAZ), 28046 Madrid, Spain; 4 Servicio de Oncología Médica, Hospital Universitario La Paz, 28046 Madrid, Spain; 5 Servicio de Cirugía General, Hospital Universitario La Paz, 28046 Madrid, Spain; 6 Servicio de Digestivo, Hospital Universitario La Paz, 28046 Madrid, Spain; 7 Centro Nacional de Investigaciones Oncológicas (CNIO), 28029 Madrid, Spain; 8 Department of Medicine and Life Sciences, Universitat Pompeu Fabra, 08003 Barcelona, Spain; 9 Departamento de Anatomía y Biología Celular, Universidad de Cantabria-IDIVAL, 39008 Santander, Spain

**Keywords:** Adherens junctions, Electron microscopy

## Abstract

Understanding the mechanisms involved in colonic epithelial differentiation is key to unraveling the alterations causing inflammatory conditions and cancer. Organoid cultures provide an unique tool to address these questions but studies are scarce. We report a differentiation system toward enterocytes and goblet cells, the two major colonic epithelial cell lineages, using colon organoids generated from healthy tissue of colorectal cancer patients. Culture of these organoids in medium lacking stemness agents resulted in a modest ultrastructural differentiation phenotype with low-level expression of enterocyte (*KLF4*, *KRT20, CA1, FABP2*) and goblet cell (*TFF2*, *TFF3*, *AGR2*) lineage markers. BMP pathway activation through depletion of Noggin and addition of BMP4 resulted in enterocyte-biased differentiation. Contrarily, blockade of the Notch pathway using the γ-secretase inhibitor dibenzazepine (DBZ) favored goblet cell differentiation. Combination treatment with BMP4 and DBZ caused a balanced strong induction of both lineages. In contrast, colon tumor organoids responded poorly to BMP4 showing only weak signals of cell differentiation, and were unresponsive to DBZ. We also investigated the effects of 1α,25-dihydroxyvitamin D_3_ (calcitriol) on differentiation. Calcitriol attenuated the effects of BMP4 and DBZ on colon normal organoids, with reduced expression of differentiation genes and phenotype. Consistently, in normal organoids, calcitriol inhibited early signaling by BMP4 as assessed by reduction of the level of phospho-SMAD1/5/8. Our results show that BMP and Notch signaling play key roles in human colon stem cell differentiation to the enterocytic and goblet cell lineages and that calcitriol modulates these processes favoring stemness features.

## Introduction

Organoids are increasingly used as a model system for many biological and disease processes, many of which are associated with altered activity of cell differentiation programs [1]. In particular, patient-derived organoids (PDO) are extensively used to dissect the characteristics and mutational landscape of colorectal tumorigenesis, as well as in large and in personalized drug activity screenings in this and other neoplasias [2–5]. However, studies on intestinal stem cell differentiation are scarce, and most of them have been performed using murine small intestine organoids [6–8]. Thus, very few studies have been reported in human colon systems: isolated crypts, in vitro immortalized normal epithelial cells, or tumor organoids and cell lines [9–13]. Moreover, a common limitation of existing studies is their restriction to the expression of marker genes of stem cells and/or differentiated cell lineages, as they usually lack a morphological analysis of cell phenotypes.

**Fig. 1:**
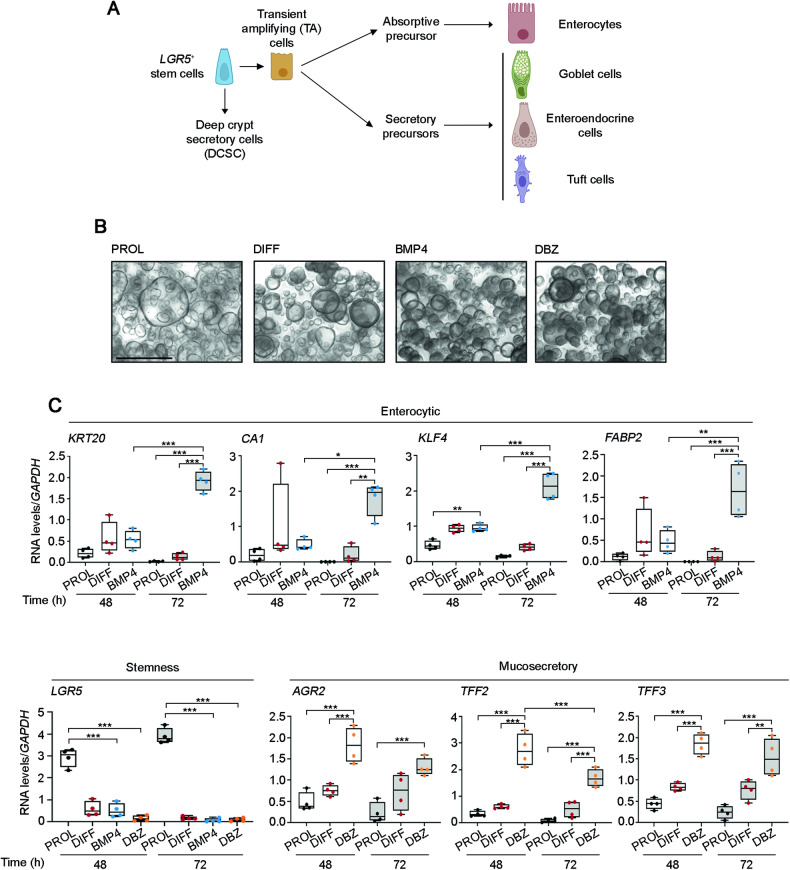
Single BMP4 or DBZ treatment respectively upregulate enterocytic and mucosecretory genes in human colon organoids. **A** Scheme of the differentiation pathways of the *LGR5*^*+*^ stem cells toward the distinct mature epithelial cell types in human colon tissue. **B** Light microscopy images of colon normal organoids cultured in PROL, DIFF, BMP4 or DBZ media for 72 h (patient #110). Scale bar, 500 μm. **C** RT-qPCR analysis of the RNA levels of stemness, enterocytic, and mucosecretory genes in organoids from patients #47, #86, #110, #130 for 48 h and #47, #110, #130, #159 for 72 h, cultured in PROL (black), DIFF (red), BMP4 (blue) or DBZ (orange) media. *P < 0.05, **P < 0.01, *** *P* < 0.001.

The best recognized marker of stem cells in the small intestine and colon is LGR5, a membrane receptor for R-spondins (RSPOs), a group of potentiators of canonical WNT signaling. During their migratory process towards the crypt top, the progeny of LGR5-expressing stem cells located at the colon crypt bottom gives rise to several precursor and later differentiated cell types: absorptive enterocytes (>80%), mucosecretory goblet cells (18%), hormone-secreting enteroendocrine cells (1%) and chemosensory and immunoregulatory Tuft cells (0.4%) (Fig. 1A) [14]. Terminal differentiation is completed in 5–7 days and is followed by cell apoptosis and debris elimination into the intestinal lumen. Stem cells also generate deep crypt secretory cells (DCSC), which are at least partially equivalent to small intestine Paneth cells. Rodent DCSC remain at the crypt bottom and last for a few weeks, having a homeostatic function on the stem cell niche providing trophic support and regenerative capacity to *lgr5*^*+*^ stem cells and possibly goblet cell maintenance [15].

Globally, data from the literature indicate that intestinal stem cell differentiation requires lack of canonical WNT signaling, stimulation of bone morphogenetic protein (BMP) and region-specific Notch pathway activity, and a poorly understood action of Hippo, Hedgehog, non-canonical WNT pathways, and EGFR ligands [16, 17]. In the murine gut, BMP2 and BMP4 are major inducers of *lgr5*^*+*^ cell differentiation by binding to their type 1 receptor (BMPR1A/alk3) and the subsequent complex and activation of BMPR2, which phosphorylates and activates BMPR1A/alk3 [18]. This, in turn, phosphorylates SMAD1/5/8 proteins, which bind SMAD4 and translocate into the cell nucleus to repress stem cell genes [19]. This process takes place only at the crypt top associated to the absence of canonical WNT factors, as myofibroblasts and smooth muscle cells surrounding the crypt bottom secrete several BMP inhibitors such as Noggin and Gremlins. Thus, a gradient of BMP activity is created along the crypt axis that controls enterocyte and goblet cell phenotype [9, 13].

Active Notch signaling at the crypt bottom, provided by membrane-bound ligands expressed by Paneth cells (and supposedly DCSC), collaborates with canonical WNT promoting self-renewal and stemness of *lgr5*^*+*^ cells. Instead, at the crypt top, Notch signaling contributes in the absence of canonical WNT activity to cell fate specification toward the enterocytic lineage, as its blockade by γ-secretase inhibitors or genetic ablation induces differentiation into secretory cells [20, 21]. Mouse and human small intestine enteroendocrine cell differentiation requires WNT and Notch inhibition and BMP2/4 activation, is repressed by ZNF800 [22] and requires quiescence caused by inhibition of EGFR and MEK [6, 23]. Differentiation of Tuft cells depends on the activation of interleukin (IL)-13 [24], while that of mouse colonic *Reg4*^*+*^ DCSC is regulated by Sprouty2 and IL-13 [15].

Vitamin D deficiency has been associated to several extraskeletal disorders including inflammatory bowel diseases and colorectal cancer [25, 26]. In line with this, the active vitamin D metabolite 1α,25-dihydroxyvitamin D_3_ (calcitriol) is a main regulator of gut physiology [27, 28]. Previously, we have described that vitamin D receptor (VDR) is expressed in *LGR5*^+^ colon stem cells in human tissue and organoids, and that calcitriol has profound gene regulatory effects in human colon organoids that are actively proliferating in growth medium containing Wnt3a and RSPO1 [29].

In this study, we sought to get further insight into the differentiation of human colon normal stem cells. To this end, and to overcome the limitations of published studies, we established a living biobank of primary PDO generated from healthy colon tissue. First, we examined the effect of single or combined treatment of these organoids with BMP4 and/or the chemical Notch inhibitor dibenzazepine (DBZ) on: a) the expression of marker genes and proteins of cell stemness and differentiation, and b) the ultrastructural cell phenotype. In addition, the response of colon tumor organoids to these treatments was investigated. Second, we analyzed the effects of calcitriol on the action of BMP4 and Notch blockade at both levels, transcriptional profile by RNA-sequencing (RNA-seq) and cell differentiation by electron microscopy analyses.

## Results

### BMP and Notch pathways modulate the expression of stemness and differentiation genes in human colon epithelial stem cells

To investigate the role of BMP and Notch pathways on the differentiation of human colon epithelial stem cells, and as an in vivo reference, we firstly analyzed the cellular composition of colon crypts in human healthy tissue by electron microscopy. Supplementary Fig. S1 shows the colon crypt morphology and the phenotype of cells located at the bottom and middle/top regions (Supplementary Fig. S1A). Precursor transit amplyfying (TA) cells and differentiated enterocytes with a microvilli-rich apical surface (Supplementary Fig. S1B), goblet cells with the apical cytoplasm full of mucin-containing secretory vesicles (Supplementary Fig. S1C), and polarized enteroendocrine cells with abundant electron-dense granules at the basal pole (Supplementary Fig. S1D) populate the middle/top region until they die by apoptosis at the tip. By contrast, the crypt bottom (Supplementary Fig. S1E) contains predominantly elongated undifferentiated stem cells (Supplementary Fig. S1F) together with some partially differentiated enteroendocrine cells (Supplementary Fig. S1G) and putative DCSC with some secretory vesicles (Supplementary Fig. S1H).

We examined the effect of BMP and Notch pathways in colon normal organoids generated from adjacent normal tissue of four colorectal cancer patients. BMP4 responsiveness was checked by analyzing the increase in phospho-SMAD1/5/8 proteins (Supplementary Fig. S2A) and the induction of the BMP4 target genes *ID2* and *DKK1* (Supplementary Fig. S2B). The effect of Notch signaling blockade was analyzed by using the γ-secretase inhibitor dibenzazepine (DBZ). DBZ activity was checked by the decrease in the expression of the Notch target gene *HES1* (Supplementary Fig. S2C). As controls we used organoids growing in proliferation medium (PROL) or in basal differentiation (DIFF) medium that lacks stemness agents.

Once the responsiveness to these two agents was confirmed, organoids were incubated for 48 h or 72 h in PROL medium, DIFF medium or in DIFF medium supplemented with BMP4 (BMP4 medium) or DBZ (DBZ medium). Organoids growing in PROL medium showed a thin-walled, monolayer cystic morphology, while a high proportion of organoids in DIFF medium presented increased thickness (Fig. 1B) that electron microscopy analysis revealed to be due to bigger cell size and/or to becoming multilayer. This effect was more pronounced in BMP4 and DBZ media. Next, we studied the expression of representative marker genes of cell stemness or the distinct differentiated intestinal cell lineages by RT-qPCR. We specifically analyzed the stemness *LGR5* gene, as in the mouse intestine loss of *lgr5* expression directly correlates with an increase in the expression of differentiation markers and lineage commitment as cells exit the niche environment [30, 31]. As shown in Fig. 1C, the level of *LGR5* RNA was much lower in organoids cultured in DIFF, BMP4 or DBZ media than in PROL medium. Contrarily, the RNA levels of the enterocytic genes *KRT20*, *CA1*, *KLF4* and *FABP2* increased slightly at 48 h and significantly at 72 h of incubation in BMP4 medium. The RNA levels of the mucosecretory genes *AGR2, TFF2*, and *TFF3* strongly increased already at 48 h of incubation in DBZ medium as compared to PROL medium. This effect was more moderate at 72 h of incubation (Fig. 1C).

**Fig. 2:**
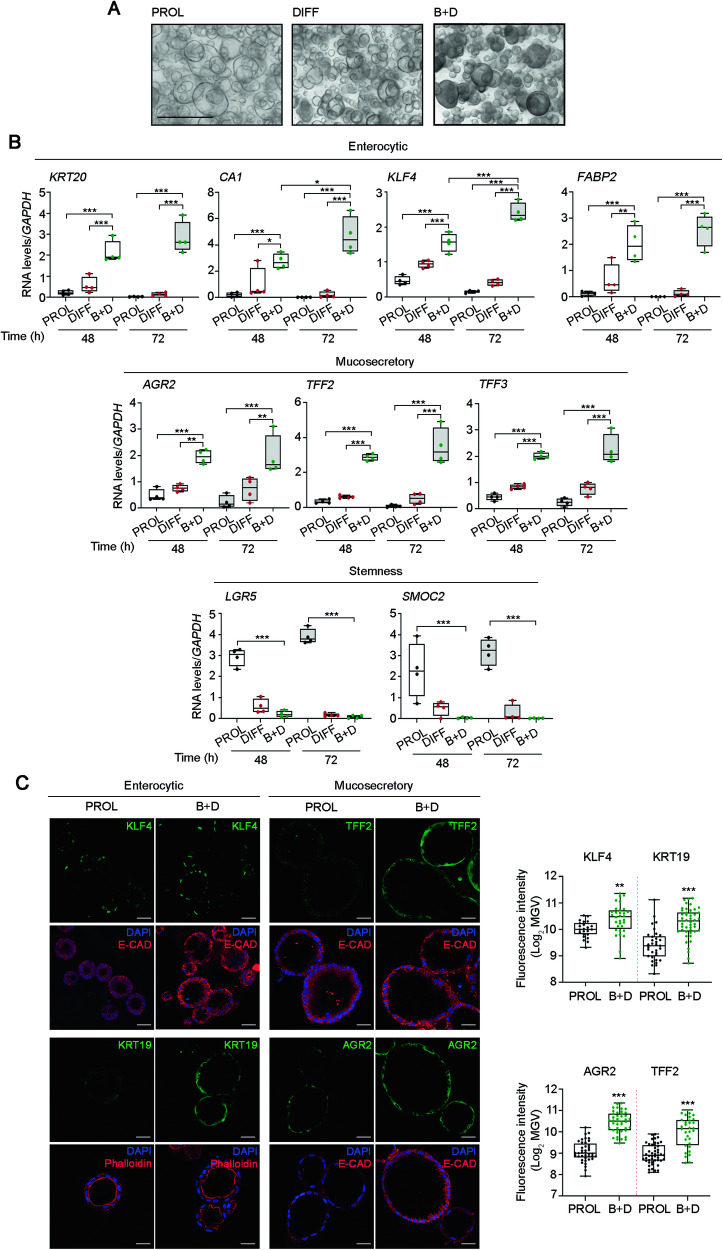
The combination of BMP4 and DBZ strongly induces the expression of enterocytic and mucosecretory markers. **A** Light microscopy images of colon normal organoids cultured in PROL, DIFF, or B + D media for 72 h (patient #110). Scale bar, 500 μm. **B** RT-qPCR analysis of the RNA levels of stemness, enterocytic and mucosecretory genes in organoids from patients #47, #86, #110, #130 for 48 h and #47, #110, #130, #159 for 72 h cultured in PROL (black), DIFF (red) or B + D (green) media. *P < 0.05, **P < 0.01, ***P < 0.001. **C** Immunofluorescence analysis of the expression of enterocytic and mucosecretory protein markers (green) in organoids cultured for 48 h in PROL or B + D media (patient #158). Scale bars, 30 µm. Box-plots show the quantification of the fluorescence intensity (Log_2_ MGV) (patients #92, #158 and #166; the number of organoids analyzed was 55 for KLF4, 80 for KRT19, 83 for AGR2 and 79 for TFF2). **P < 0.01, ***P < 0.001. MGV: mean gray value.

These results prompted us to study the effect of the combination of BMP4 and DBZ. The incubation in DIFF medium enriched with both agents, BMP4 and DBZ (B + D medium), led to the formation of dense, thick-walled organoids (Fig. 2A) and to the drastic inhibition of the stemness genes *LGR5* and *SMOC2* (Fig. 2B). Of note, the expression of both enterocytic and mucosecretory genes was strongly induced in B + D medium at 48 h and 72 h (Fig. 2B). These results were confirmed at the protein level by immunofluorescence analyses, which showed the increase of enterocytic (KLF4, KRT19) and mucosecretory (TFF2, AGR2) proteins upon incubation in B + D medium (Fig. 2C). Likewise, the expression of the key intercellular adhesion protein E-cadherin was higher in B + D medium than in PROL medium (Fig. 2C). Together, these results suggested the existence of a strong pro-differentiation effect of the BMP4 and DBZ combination.

### BMP and Notch pathways control colon stem cell differentiation

**Fig. 3:**
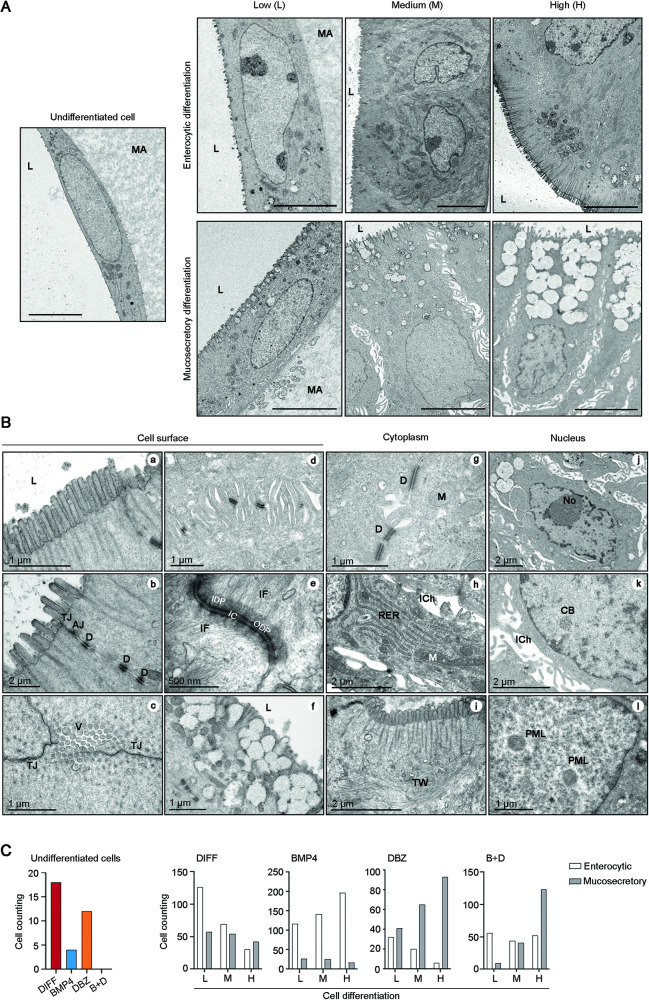
Single and combined BMP4 and DBZ treatment induce epithelial stem cell phenotypic differentiation in human colon normal organoids. **A** Representative ultrastructural images of an undifferentiated stem cell (left panel) and of cells displaying low (L), medium (M), or high (H) grade of enterocytic or mucosecretory cell differentiation in organoids incubated in DIFF, BMP4, DBZ or B + D media for 48 h. Scale bars, 5 μm. L, lumen; MA, Matrigel. **B** Electron microscopy differentiation features of the cell surface, cytoplasm, and nucleus upon 48 h incubation: **a** microvilli; **b** intercellular adhesion structures: tight junctions (TJ), adherens junctions (AJ) and desmosomes (D); **c** tangential section of the apical pole of an enterocyte showing microvilli (V) and tight junctions (TJ); **d** cell membrane interdigitations stabilized by desmosomes; **e** high-magnification desmosomal structures: intercellular cleft (IC) with its transmembrane connecting proteins, inner and outer cytoplasmic dense plaques (IDP, ODP) and large bundles of cytokeratin intermediate filaments (IF) attached to the plaques; **f** docking of mucin-secretory vesicles to cell membrane at the apical pole of a goblet cell; **g** mitochondria (M) and desmosomes (D); **h** rough endoplasmic reticulum (RER) cisterns and intercellular chambers (ICh); **i** terminal web (TW) of cytokeratin intermediate filaments; **j** nucleus with a prominent nucleolus (No) and numerous masses of heterochromatin; **k** Cajal body (CB) free in the nucleoplasm and intercellular chambers (ICh); **l** PML bodies. **C** Left, number of undifferentiated cells in each medium (DIFF, red; BMP4, blue; DBZ, orange; and B + D, green). Right, quantification of the number of cells displaying L, M, or H enterocytic (white) or mucosecretory (gray) differentiated phenotype of organoids cultured in the indicated media (patients #47, #86, #92). A total of 1520 cells were analyzed: 396, 530, 269 and 325 cells in DIFF, BMP4, DBZ and B + D media, respectively.

Based on the observed induction of genes that are characteristic of mature enterocytes and goblet cells, we next explored the effects of BMP4 and DBZ on the cell phenotype in colon organoids. Ultrastructural analysis of organoids from three patients incubated in PROL medium showed an undifferentiated cell phenotype: (i) elongated cell shape, (ii) predominance of euchromatin, (iii) absence of microvilli and intercellular adhesion structures, iv) abundant free ribosomes, and (v) paucity of cytoplasmic organelles (Fig. 3A). On the contrary, upon 48 h incubation in DIFF, BMP4, DBZ or B + D media, the majority of cells in the organoids showed variably advanced structural hallmarks of differentiated enterocytic or mucosecretory lineages (Fig. 3A). At a higher magnification, enterocytic differentiation features at the cell surface included (i) long and densely arranged microvilli with deep cytoplasmic rootlets of actin microfilaments (Fig. 3Ba, i), (ii) adhesion structures such as tight junctions, adherent junctions, abundant desmosomes and interdigited cell junctions (Fig. 3Ba–e), and (iii) other lateral cell surface specializations such as dilated intercellular chambers (Fig. 3Bh, k). The main mucosecretory differentiation characteristic was the apical accumulation of clusters of electron-lucent mucin-secretory vesicles, some of them docking to plasma membrane domains usually surrounded by short microvilli (Fig. 3Bf). Cytoplasmic differentiation features common to enterocytic and mucosecretory lineages included the presence of abundant organelles (mitochondria, rough endoplasmic reticulum cisterns) (Fig. 3Bg, h), while the formation of compact subcortical bundles of cytokeratin intermediate filaments (terminal web) that function as anchors for the actin cytoskeleton of microvilli was specific of enterocytes (Fig. 3Bi). Also, a common feature to both differentiated cell lineages was the presence of irregularly shaped nuclei (frequently lobulated and with nuclear envelope invaginations) containing heterochromatin patches and structures such as Cajal bodies and PML bodies (Fig. 3Bj–l).

We performed a morphometric and quantitative ultrastructural analysis to classify the grade of cell differentiation in three categories: low (L), medium (M) or high (H) (Fig. 3A). Given that the polymerization and bundling of a core of actin filaments into the microvilli and rootlets of the brush border is the main factor in the enterocyte differentiation [16, 32], we used these ultrastructural features: (i) the number of microvilli/µm width of the luminal cell border (microvilli density), and (ii) the rootlets length (µm) (Supplementary Table S1). “Microvillus” is defined as the segment of a core bundle of actin filaments that is covered by the plasma membrane, and “rootlet” as the segment free of membrane extending into the apical cytoplasm. L differentiated enterocytes lack rootlets and their microvilli were less dense than in M and H differentiated cells (Fig. 3A). M differentiated cells show microvilli with short rootlets, whereas H differentiated cells exhibit long, densely packed and ordered microvilli with deep intracytoplasmic rootlets (Fig. 3A). Regarding goblet cell differentiation, it is classically known that immature goblet cells contain few secretory vesicles, whereas mature goblet cells exhibit large accumulations of vesicles which aggregate into clusters [33]. Consequently, we used the following ultrastructural parameters: i) the number of clusters of secretory vesicles per cell profile, and ii) the cytoplasmic area occupied by these clusters (µm^2^) (Supplementary Table S1). L differentiated goblet cells have a reduced number of secretory vesicles and basically lack aggregation into clusters as compared to M differentiated goblet cells, which present few clusters covering small cytoplasmic areas, and H differentiated goblet cells that have many clusters that cover bigger areas of the supranuclear cytoplasm (Fig. 3A).

On the basis of the parameters indicated above, the specific criteria shown in Supplementary Table S1 were selected for the ultrastructural classification of the grade of enterocyte and goblet cell differentiation. We counted cells that displayed L, M or H graded phenotype in series of electron microscope micrographs from organoids of three patients incubated in each culture medium. All counts are compiled in Supplementary Table S2 and a graphic representation of the number of cells according to the grade of each differentiation lineage is shown in Fig. 3C. The distinct distribution of graded differentiated cells in organoids incubated in the four media was statistically significant (*P* < 0.001). In DIFF medium, 18/396 (5%) cells remained undifferentiated, while the rest displayed preferentially low enterocytic differentiation (Fig. 3C). In contrast, in BMP4 medium only 4/530 (1%) cells were undifferentiated while most cells displayed an enterocytic phenotype that was in a large proportion highly differentiated, and the number of cells with a mucosecretory phenotype was very low (Fig. 3C). Conversely, in DBZ medium the majority of cells acquired mucosecretory differentiation while only a few of them showed high enterocytic differentiation (Fig. 3C), and the proportion of undifferentiated cells was 12/269 (4%). Interestingly, in B + D medium no cells remained undifferentiated and the distribution of cells showing differentiation toward the enterocytic and mucosecretory pathways was balanced, though with higher proportion of cells showing an advanced mucosecretory phenotype (Fig. 3C). Remarkably, the analysis of organoids at low magnification revealed the presence of three types of organoids in B + D differentiation media: 15% showing homogenous enterocytic differentiation, 20% showing homogenous mucosecretory phenotype, and 65% mixed, containing differentiated cells of both lineages (Supplementary Fig. S3).

**Fig. 4:**
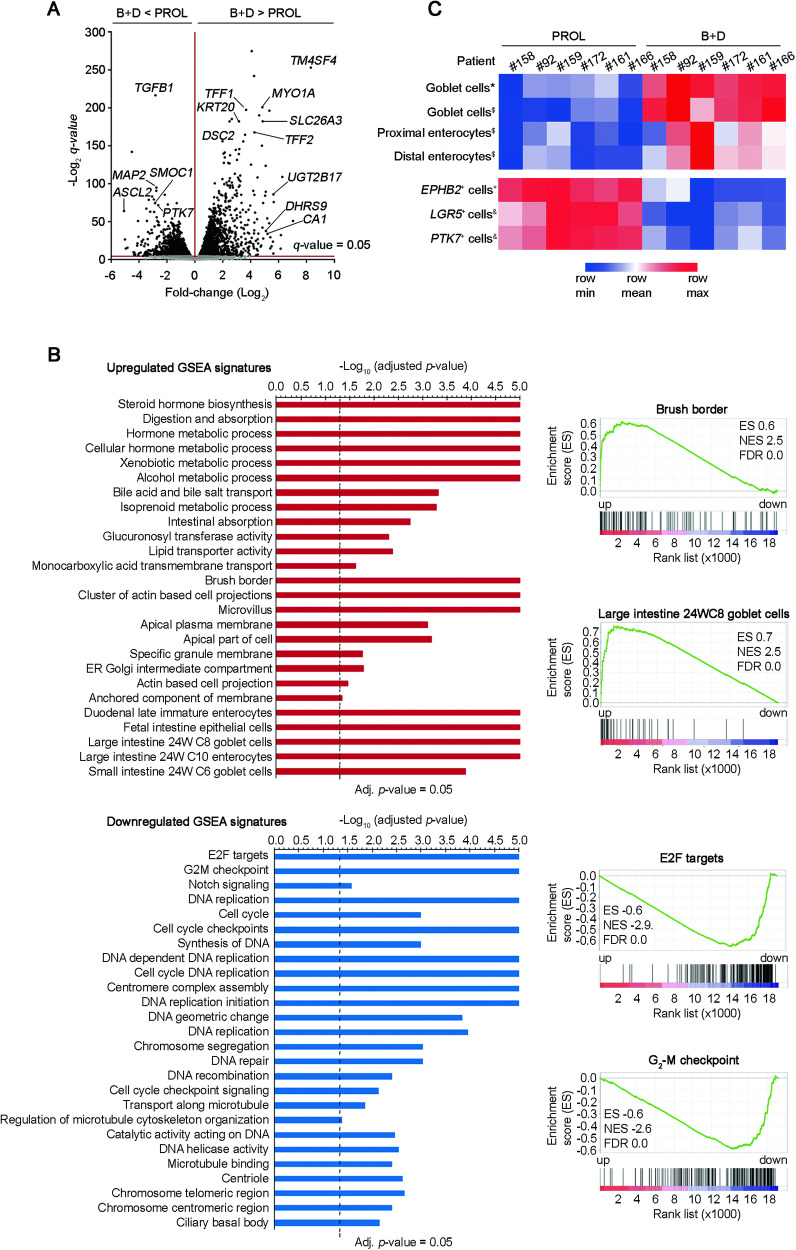
Combined BMP4 and DBZ treatment induces transcriptomic differentiation signatures in human colon normal organoids. **A** Volcano plot comparing RNA-seq signatures from six organoid cultures (patients #92, #158, #159, #161, #166, and #172) for 48 h in PROL or B + D media. The x-axis shows the fold-change (Log_2_) and the y-axis shows the *q*-value (−Log_2_). Each dot represents a gene. Dots above the line were significant. **B** Left, significant regulated GSEA signatures associated with gene expression profile of organoids cultured in B + D medium; right, GSEA comparing two differentiated-related signatures (upper graphs) and two proliferation-related signatures (lower graphs) with the organoids cultured in B + D medium. ES, enrichment score; NES, normalized enrichment score; FDR, false discovery rate. **C** Heatmap showing the homology of RNA-seq organoids cultured in PROL or B + D media with differentiated epithelial intestinal or stemness signatures. ^*^ [55], ^$^ [56], ^+^ [57], ^&^ [58].

To further characterize the differentiation process induced by the incubation in B + D medium, we performed RNA-seq assays. Colon normal organoids from six patients were cultured in PROL or B + D media (48 h). Their transcriptomic profiles showed statistically significant differentially expressed genes: as compared to PROL medium, in B + D medium stemness genes such as *SMOC2*, *ASCL2, LGR5, MEX3A*, and *PTK7* were downregulated, while all differentiation genes previously studied by RT-qPCR were upregulated (Fig. 4A and Supplementary Table S3). The expression of marker genes for enteroendocrine cells (*CHGA*) or Tuft cells (*PTGS1*) was hardly detectable in PROL medium, while that of DCSC cells (*REG4*) was quite low. In B + D medium, only *REG4* RNA expression increased significantly (Supplementary Table S3). Since in mouse small intestine organoids enteroendocrine cell differentiation requires quiescence [6], we examined this possibility in our system. Indeed, we detected the upregulation of *CHGA* RNA expression in the absence of EGF and Wnt3a and in the presence of a MAPK inhibitor; however, this treatment was cytotoxic and caused organoid collapse (not shown).

Gene set enrichment analyses (GSEA) confirmed that organoids cultured in B + D medium displayed high expression of a large number of intestinal differentiation signatures and downregulation of DNA synthesis and cell proliferation signatures (Fig. 4B). Concordantly, single sample GSEA (ssGSEA) clustered the organoid cultures according to previously described stemness or differentiated gene sets. The heatmap showed an enrichment of enterocytic and goblet cell gene sets in organoids cultured in B + D medium and, contrarily, *LGR5* and *PTK7* stemness and bottom crypt *EPHB2* signatures where enriched in organoids cultured in PROL medium (Fig. 4C).

### Calcitriol modulates gene expression during the differentiation of colon epithelial cells

We sought to investigate the effect of calcitriol on the differentiation capacity of colon normal stem cells. To this end, we first studied whether organoids were responsive to calcitriol. As shown in Supplementary Fig. S4A, most PDO expressed comparable RNA levels of *VDR*, the high affinity vitamin D receptor. Accordingly, *CYP24A1*, the most characteristic calcitriol target gene was strongly upregulated (Supplementary Fig. S4B).

**Fig. 5:**
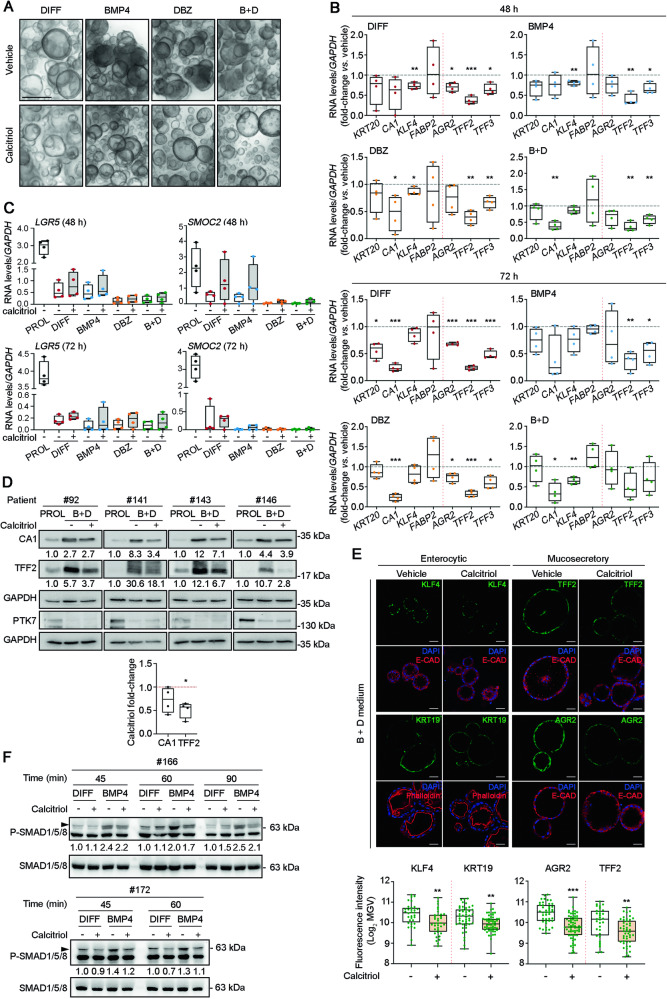
Calcitriol antagonizes BMP4 and DBZ-mediated differentiation in human colon normal organoids. **A** Light microscopy images of organoids (patient #110) cultured for 72 h in DIFF, BMP4, DBZ or B + D media in the absence (vehicle) or presence of calcitriol (100 nM). Scale bar, 500 μm. **B** RT-qPCR analysis of the RNA levels of enterocytic (*KRT20*, *CA1*, *KLF4* and *FABP2*) and mucosecretory (*AGR2*, *TFF2* and *TFF3*) genes in organoids from patients #47, #86, #110, #130 for 48 h and #47, #110, #130, #159 for 72 h cultured in DIFF (red), BMP4 (blue), DBZ (orange) or B + D (green) media in the absence (vehicle) or presence of calcitriol (100 nM). *P < 0.05, **P < 0.01, ***P < 0.001. **C** RT-qPCR analysis of the RNA levels of stemness genes in organoids from the same patients and in the same conditions as in B. **D** Western blot analysis and quantification of the effect of calcitriol on the expression of enterocytic (CA1) and mucosecretory (TFF2) marker proteins in organoids from four patients incubated in PROL or B + D media in the absence (vehicle) or presence of calcitriol (100 nM) for 48 h. The stemness PTK7 protein was used as control of differentiation and GAPDH as loading control. *P < 0.05. **E** Immunofluorescence analysis of the expression of enterocytic and mucosecretory protein markers in organoids (patient #158) cultured for 48 h in PROL or B + D media in the absence (vehicle) or presence of calcitriol (100 nM). Scale bars, 30 µm. Box-plots show the quantification of fluorescence intensity (Log_2_ MGV) (patients #92, #158 and #166; the number of organoids analyzed was 68 for KLF4, 104 for KRT19, 110 for AGR2 and 79 for TFF2). **P < 0.01, ***P < 0.001. **F** Western blot analysis and quantification of the level of phospho(P)-SMAD1/5/8 in organoid cultures that were incubated for 24 h with calcitriol (100 nM) or vehicle before incubation in DIFF medium or BMP4 medium (50 ng/mL) during the indicated times. MGV: mean gray value.

Next, organoids from four patients were incubated in DIFF, BMP4, DBZ or B + D media in the presence or absence of calcitriol. After 72 h, visual inspection under the light microscope revealed that calcitriol reduced partially the formation of thick organoids induced by culture in all four media, which suggested an effect on the process of cell differentiation (Fig. 5A). In line with this, the expression of enterocytic and mucosecretory genes was variably inhibited by calcitriol at both 48 h and 72 h of incubation in the four media (Fig. 5B). Concordantly, in all media the addition of calcitriol caused a tendency to an increased expression of the stemness genes *LGR5* and *SMOC2* (Fig. 5C). At the protein level, calcitriol reduced the induction of enterocytic and mucosecretory differentiation markers (Fig. 5D,E) while, as expected, the stemness PTK7 marker decreased in B + D medium (Fig. 5E).

To investigate whether the repression of the pro-differentiation action of BMP4 by calcitriol could result from the direct inhibition of BMP4 early signaling, PDO cultured in PROL medium were preincubated with calcitriol or vehicle for 24 h and changed to DIFF medium supplemented or not with BMP4. Western blot analysis showed that calcitriol consistently attenuated the rapid increase in the level of phospho-SMAD1/5/8 induced by BMP4 (Fig. 5F).

**Fig. 6:**
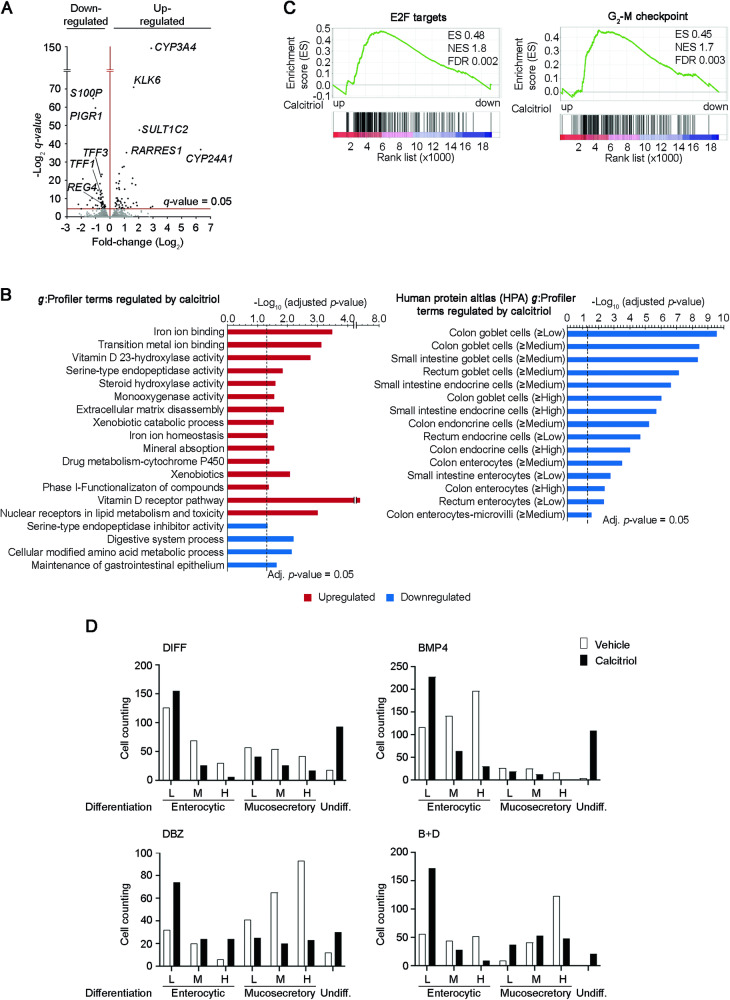
Calcitriol attenuates stem cell phenotypic differentiation in human colon normal organoids. **A** Volcano plot comparing RNA-seq signatures from six organoid cultures (patients #92, #158, #159, #161, #166 and #172) during 48 h in PROL or B + D media in the absence (vehicle) or presence of calcitriol (100 nM). The x-axis shows the fold-change (Log_2_) and the y-axis shows the *q*-value (-Log_2_). Each dot represents a gene. Dots above the line were significant. Genes regulated by calcitriol are indicated. **B** *g*:Profiler functional enrichment studies show functional regulated terms (left) and Human Protein Atlas profiles (right) significantly regulated by calcitriol in normal organoids cultured in B + D medium for 48 h. **C** GSEA comparing two proliferation-related signatures and the RNA-seq transcriptomic profiles of normal organoids cultured in B + D medium in the presence or absence of calcitriol. **D** Quantification of the number of cells displaying low (L), medium (M) or high (H) enterocytic or mucosecretory differentiated phenotype in normal organoids cultured in DIFF, BMP4, DBZ or B + D media in the absence (white) or presence (black) of calcitriol (100 nM) for 48 h (patients #47, #86, #92). We analyzed a total of 1520 cells treated with vehicle (396, 530, 269 and 325 cells in DIFF, BMP4, DBZ and B + D media, respectively), and 1418 cells treated with calcitriol (364, 466, 220 and 368 cells in DIFF, BMP4, DBZ and B + D media, respectively).

To better characterize calcitriol action, we examined the transcriptomic profile of organoids cultured in B + D medium in the presence or absence of calcitriol for 48 h. RNA-seq showed that calcitriol upregulates genes mainly involved in a variety of metabolic routes while it downregulates genes linked to colon enterocytic, enteroendocrine and mucosecretory cell differentiation, and it also reduces the RNA expression of the DCSC marker *REG4* gene (Fig. 6A, B and Supplementary Table S4). Accordingly, GSEA revealed that the growth arrest characteristic of differentiated cells (Fig. 4B) was reverted by calcitriol treatment, that induces transcriptomic signatures associated to cell proliferation such as E2F targets and G_2_-M checkpoint (Fig. 6C).

### Calcitriol modulates cell phenotype and viability in differentiating colon normal organoids

To examine whether the effects of calcitriol on gene expression were reflected at the cell phenotype, we performed an ultrastructural analysis of normal organoids cultured in DIFF, BMP4, DBZ, or B + D media containing calcitriol. The comparison of the results of this analysis with those obtained previously in the absence of calcitriol (Fig. 3C) showed that calcitriol altered significantly (*P* < 0.001) the distribution of cells in the media (Fig. 6D). Thus, calcitriol augmented the proportion of undifferentiated cells and reduced advanced differentiation toward both enterocytic and mucosecretory cell lineages, with the exception of enterocytic differentiation in DBZ medium (Fig. 6D and Supplementary Table S2).

Calcitriol inhibits cell proliferation in many culture systems and also in PDO incubated in growth medium (PROL) [29, 34]. As cell proliferation and differentiation are usually opposite processes, a nearly complete absence of mitotic cells was found upon change of colon normal organoids to the pro-differentiation B + D medium (not shown). Moreover, advanced/terminal differentiation to either enterocytic or goblet cell lineages was followed by cell degeneration and death, as it happens in other in vitro systems such as differentiating PC12 pheochromocytoma cells or F9 teratocarcinoma stem cells [35, 36]. Thus, we examined whether calcitriol could affect cell viability in normal organoids incubated in differentiation media. In line with its effect attenuating cell differentiation, calcitriol extended cell viability particularly in organoids long-term incubated in BMP4 and B + D media (Supplementary Fig. S5).

### Colon tumor organoids do not differentiate in response to BMP and Notch modulation

**Fig. 7:**
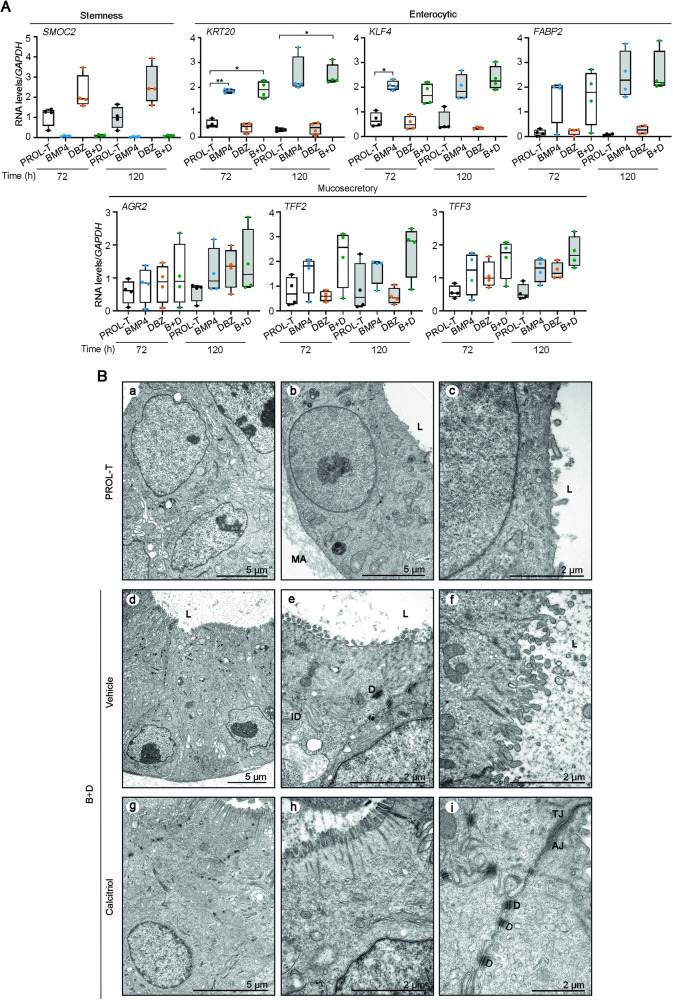
BMP4, DBZ and B + D media fail to induce cell differentiation in human colon tumor organoids. **A** RT-qPCR analysis of the RNA levels of stemness, enterocytic, and mucosecretory genes in tumor organoids from patients #30, #48, #53 and #66 cultured in PROL-T (black), BMP4 (blue), DBZ (orange) or B + D (green) media for 72 h or 120 h. *P < 0.05, **P < 0.01. **B** Ultrastructural images of tumor organoids incubated for 72 h in PROL-T medium: **a** large nuclei with predominance of euchromatin, **b** euchromatic nucleus with prominent nucleolus, and smooth cell surface at the apical pole, **c** apical cell surface with isolated, rudimentary microvilli. In B + D medium in the absence of calcitriol: **d** prismatic polarized cells with irregular shaped nuclei, small aggregates of heterochromatin at the nuclear periphery and apical microvilli, **e** immature and disorganized microvilli and presence of few desmosomes (D) and interdigitations (ID), **f** higher magnification of the apical cell surface illustrating very irregular and immature microvilli without rootlets. In B + D medium in the presence of calcitriol (100 nM): **g** a slightly differentiated cell, **h** mature microvilli with their rootlets, **i** prominent intercellular adhesion structures, including a tight junction (TJ), an adherens junction (AJ) and numerous desmosomes. L lumen, MA Matrigel.

A published global transcriptomic study performed in proliferative conditions revealed that tumor organoids from colorectal cancer patients expressed higher levels of stemness genes and lower levels of differentiated enterocytic and mucosecretory markers than their matched normal organoids [29]. To investigate whether cancer stem cells present in colon tumor organoids could respond to differentiation stimuli, we studied the expression of marker genes in organoid cultures of four patients upon 72 h or 120 h incubation in PROL-T, BMP4, DBZ or B + D media. As shown in Fig. 7A, both the downregulation of *SMOC2* stemness gene and the upregulation of enterocytic differentiation genes indicated that tumor organoids were sensitive to BMP4 action (BMP4 and B + D media), while DBZ treatment failed to induce any mucosecretory gene. However, electron microscopy analysis revealed that these transcriptomic changes did not translate into relevant alterations of cell phenotype in tumor organoids incubated in B + D medium for 72 h (Fig. 7B). In their proliferation medium (PROL-T), tumor organoids showed a compact, usually lumen-free and sometimes multilayer cell morphology. Cells displayed an undifferentiated phenotype: large nuclei with predominance of transcriptionally active euchromatin (Fig. 7Ba), smooth cell surface or with very rudimentary and scattered microvilli (Fig. 7Bb, c), general paucity of cytoplasmic organelles, particularly rough endoplasmic reticulum cisterns, abundance of free ribosomes and reduced intercellular spaces (Fig. 7Ba, b). Contrarily to normal organoids, most cells in tumor organoids remained undifferentiated in B + D medium. Only a minor proportion of tumor cells displayed some weak enterocytic differentiation features: presence of prismatic polarized cells (Fig. 7Bd), irregularly shaped nuclei with a peripheral layer of heterochromatin (Fig. 7Bd, e), narrow intercellular chambers, and some sparse clusters of immature and disorganized microvilli (Fig. 7Bf). Of note, concordantly to the lack of effects on the RNA expression of mucosecretory marker genes, no hints of mucosecretory differentiation were found. Later than 72 h incubation, tumor organoids in B + D medium developed a progressive process of cell degeneration that ended in apoptosis (not shown).

### Effects of calcitriol on colon tumor organoids

To investigate whether calcitriol could modulate cell differentiation in colon tumor organoids cultured under differentiation conditions, we first studied the expression of marker genes in organoid cultures of four patients upon 72 h or 120 h incubation in PROL, BMP4, DBZ or B + D media. Calcitriol did not significantly change the RNA level of the enterocytic or mucosecretory genes studied under any culture condition (not shown). Interestingly and in contrast to what was found in normal organoids treated with calcitriol, the ultrastructural analysis by electron microscopy revealed a limited pro-differentiation effect of calcitriol. Thus, in tumor organoids from four patients cultured in B + D medium calcitriol promoted the formation in a subset of cells of more mature microvilli (in terms of abundance, alignment, and presence of rootlets), increased intercellular adhesion structures (tight junctions, adherens junctions and desmosomes) and plasma membrane interdigitations, and induced moderate heterochromatinization (Fig. 7Bg–i).

## Discussion

In this study, we describe the establishment of a differentiation system in PDO in which *LGR5*^*+*^ colon epithelial stem cells differentiate to the two major epithelial cell lineages, absorptive enterocytes and mucosecretory goblet cells, in response to combined activation of the BMP pathway and inhibition of Notch signaling. Our results indicate a major role of BMP4 promoting enterocytic differentiation and a strong repressive action of Notch signaling on the mucosecretory pathway. In addition, we show that calcitriol favors the maintenance of cell stemness in the organoids and attenuates the enterocytic differentiation induced by BMP4 and also the positive action of Notch inhibition on the mucosecretory differentiation pathway. Curiously, calcitriol opposes the negative effect of Notch inhibition on enterocytic differentiation.

We report that BMP4 strongly represses the stemness *LGR5* gene and upregulates genes encoding enterocytic markers and also, but less prominently, genes that are typically expressed by goblet cells in PDO cultured in medium lacking its inhibitor Noggin, Wnt3a and other stemness agents. In agreement with data in mouse systems, chemical inhibition of Notch signaling increases the expression of mucosecretory goblet cell markers in human colon organoids, while concordantly it blocks *LGR5* expression. Our data show that the co-incubation with BMP4 and the Notch inhibitor DBZ causes a stronger induction of both enterocytic and goblet cell genes and a profound change in the global transcriptomic profile as assessed by RNA-seq assays. Importantly, this cooperation between BMP activation and Notch inhibition translates into the cell phenotype: the combined treatment changes the morphology of organoids under the light microscope increasing their thickness, and it promotes the ultrastructural changes characteristics of terminal enterocytic and goblet cell differentiation as assessed by electron microscopy analysis. A careful graded quantification of cell phenotype revealed that while BMP4 alone strongly promotes the enterocytic differentiation and DBZ favors goblet cell differentiation, the combination of BMP4 and DBZ has a more balanced pro-differentiation effect toward both cell lineages, with predominance of highly differentiated mucosecretory cells.

Contrarily, all tumor organoids analyzed, that harbor a constitutively activated canonical WNT pathway (Supplementary Table S5), are mostly unresponsive in terms of cell differentiation to the combined treatment with BMP4 and DBZ. This is in conflict with the report that BMP4 has antitumor activity based on the induction of differentiation, apoptosis and chemosensitization of proposed CD133^+^ stem cells present in human colorectal tumors [37]. This apparent discrepancy is probably due to the doubtful identification of CD133^+^ cells as cancer stem cells in the referred study. Our data show that signaling cues such BMP4 that promote cell differentiation in colon normal organoids do not have similar effects on colon cancer organoids, supporting the notion that oncogenesis is tightly associated to the suppression of cell differentiation [38]. This is probably related to the constitutive activation of canonical WNT signaling found in tumor organoids, as it is well known that the removal of WNT signals is required for colon normal organoid differentiation [39].

BMP pathway is essential for the morphogenesis of multiple organs during ontogenesis and maintain tissue homeostasis postnatally by the modulation of the proliferation and differentiation of many cell types [40]. In homeostasis, BMP4 downregulates proliferation genes and activates Notch signaling only in the upper crypt region of the mouse small intestine, while this BMP and Notch interaction is responsible for the induction of epithelial-to-mesenchymal transition genes in mesenchymal-subtype colorectal tumors [11]. Curiously, it has been reported that BMP4 induces Notch signaling via SMAD5 in a γ-secretase-independent interaction, and that both pathways cooperate in a human colon epithelial cell line immortalized in vitro by expression of *CDK4* and *TERT* [10]. Surprisingly, in contradiction with our results the authors of this study state that primary human intestinal organoids collapse following BMP ligand stimulation [11].

Our findings highlight the critical role of BMP and Notch pathways in the regulation of differentiation in colon normal organoids. However, it is possible that additional pathways contribute to this process including Hedgehog and Hippo, non-canonical WNTs, EGFR ligands, and possibly other factors and cytokines that individually or by interacting in a combinatorial way may modulate the action of BMP and Notch. The activation of these pathways by ligands secreted in vivo by (myo)fibroblasts, immune and endothelial cells is hampered in our study due to the pure epithelial nature of the organoid system used. This limitation, however, does not affect much to the main conclusions of the study: the key role of the combination of BMP activation and WNT and Notch inhibition for terminal differentiation of human colon stem cells to the enterocytic and goblet cell lineages, and the unresponsiveness of colorectal tumor organoids to these pro-differentiation signals.

Vitamin D (cholecalciferol) is a major physiological regulator of the homeostatic health. Upon its synthesis in the skin by action of solar radiation or its acquisition *via* diet, the biologically inactive cholecalciferol undergoes two hydroxylation steps, the first in the liver and the second in the kidney and many epithelial and immune cells, to render the active metabolite 1α,25-dihydroxyvitamin D_3_ or calcitriol. By binding to its high affinity receptor VDR, a transcription factor of the superfamily of nuclear receptors, calcitriol modulates the expression of hundreds of genes in a cell type- and context-dependent manner [34]. Calcitriol controls the proliferation, metabolism and function of many types of normal cells and promotes the reversion of a high number of cancer cell types to a more differentiated phenotype [26, 41]. Particularly, calcitriol induces colon cancer cell differentiation in vitro supporting an antitumoral effect [42–44].

Contrarily to these notions, we show here that calcitriol opposes the pro-differentiation action of BMP4 and DBZ in colon normal organoids, thus favoring the maintenance of the normal stem cell phenotype. This result agrees with our previous observation in organoids incubated in PROL medium, in which calcitriol reduces cell proliferation and upregulates stemness genes [29]. In line with our data, Peregrina and colleagues have previously found that stem cell properties are compromised in the small intestine and colon of mice fed with a low vitamin D and calcium diet or harboring *Vdr* deletion in intestinal stem cells [45]. However, in striking contrast, Sittipo and colleagues have recently reported that calcitriol inhibits stemness and promotes differentiation and apoptosis in mouse small intestine organoids [46]. The reason for this discrepancy may be related to differences in species, intestinal segment, and /or culture conditions.

We show that calcitriol interferes with the effects of BMP4 on PDO acting at a very early step in its signaling pathway, as it inhibits the rapid accumulation of phospho-SMAD1/5/8 proteins, key effectors mediating intracellular BMP4 action. Since VDR, the unique high affinity calcitriol receptor, is a transcription factor located predominantly within the nucleus of many cell types including human colon PDO cells [29], the inhibitory effect of ligand-activated VDR on the phosphorylation of SMAD1/5/8 proteins is probably mediated by one or more calcitriol/VDR target genes. A candidate mediator is SMOC2, an inhibitor of BMP signaling in several biological systems [47–49], that is upregulated by calcitriol in human colon PDO through binding of VDR to the *SMOC2* gene promoter [29]. As for the calcitriol and Notch cross-talk, the complexity of the Notch pathway (ligands, receptors) and the large variability of their interaction in different biological systems hinders pointing to a specific candidate mechanism. A recent study has reported that vitamin D (supposedly calcitriol) decreases NOTCH1 protein expression in human SW480 colon carcinoma cells [50]. However, data from organoids and carcinoma cell lines are usually not coincident.

This study reveals novel unprecedented actions of vitamin D/calcitriol through the regulation of colon stem cell pools and function that may be crucial in vivo for intestinal homeostasis; aging, by preventing stem cell depletion/exhaustion during lifetime; and also upon injury, potentially protecting from chemotherapy-induced damage or other insults. In line with this, *Vdr* has recently been included in a stemness signature of mouse intestinal stem cells [51] and vitamin D deficiency has been proposed as a risk factor for accelerated brain aging and several age-related diseases [52, 53]. Finally, our results from colon tumor organoids suggest that the proposed protective action of vitamin D against colorectal cancer raised from data of many epidemiological and preclinical studies, might to a certain extent result from non-cancer cell autonomous effects, possibly through actions on the tumor microenvironment (CAFs, endothelial cells….) and on the immune system.

In conclusion, the cell differentiation system here presented will be useful for the analysis of the action of agents and signals on the human colon epithelium and relevant for the study of gut alterations including colon cancer and inflammatory bowel diseases (ulcerative colitis and Crohn’s disease).

## Materials and methods

### Human samples

Fresh human tissues were provided by Biobank IdiPAZ (PT20/0000), integrated into the Spanish Biobank Network (www.redbiobancos.es). Biopsies were obtained from individuals diagnosed with colorectal cancer and subjected to surgery or endoscopic procedure between 2014 and 2023. Patient data and mutational status of the tumor organoids are summarized in Supplementary Table S5. Normal tissue samples were obtained from the area distant to the tumor and the histology of the biopsies was evaluated by the pathology services of Hospital Universitario La Paz (Madrid). All human subjects gave informed consent.

### Organoids isolation, culture and passage

Establishment of colon normal and tumoral organoid culture was performed as previously described [29]. Briefly, human colon normal biopsies were incubated with a mixture of antibiotics (Primocin [Invivogen, CA, USA], Gentamycin and fungizone [Thermo Fisher Scientific, MA, USA]) for at least 1 h in rotation at room temperature (RT). Then, crypts were extracted by incubating the biopsies, first with 10 mM dithiothreitol (DTT, Tocris, Bristol, UK) for 5 min at RT, and later with 8 mM EDTA solution for 5 min at RT and 60 min in slow rotation at 4 °C. Crypts were then washed in PBS and washing buffer (Advanced DMEM/F12, 10 mM HEPES, and 10 mM Glutamax [Thermo Fisher Scientific]). Finally, crypts were embedded in Matrigel (Corning, NY, USA) and seeded in drops on pre-warmed 6-well plates. After Matrigel solidification, the proliferation medium (PROL) was added, that is composed of 50% Advanced DMEM/F12, 50% L-WRN-conditioned medium (from L-WRN cells [ATCC CRL3276] secreting Wnt3a, Noggin and RSPO3), 10 mM HEPES, 10 mM Glutamax, 10 mM Nicotinamide (Sigma-Aldrich, MD, USA), 1×N2 supplement (Thermo Fisher Scientific), 1×B27 supplement (Thermo Fisher Scientific), 1 mM N-acetyl-L-cysteine (Sigma-Aldrich), 1:500 Primocin, 0.1 μg/mL Noggin (Peprotech, London, UK), 1 μg/mL Gastrin (Tocris), 1 μg/mL RSPO1 (Sino Biological, Beijing, China), 50 ng/mL EGF (Peprotech), 0.02 μM PGE_2_ (Sigma-Aldrich), 1 μM LY-2157299 (Axon-Medchem, Groningen, The Netherlands), and 10 μM SB-202190 (Sigma-Aldrich).

Tumor biopsies were incubated with the same mixture of antibiotics than normal biopsies. Cancer stem cells were released by digesting enzymatically the biopsy with 1 mg/mL type IV collagenase (Sigma-Aldrich) in PBS for 30 min at RT. Cell suspension was passed through a 18 G syringe with a later filtration using a 200 µm mesh filter. Finally, single cells were embedded in Matrigel and plated on pre-warmed 12-well dishes. Proliferation medium of tumor organoids (PROL-T) was PROL medium lacking L-WRN conditioned medium, RSPO1, and Nicotinamide. To prevent anoikis, 6 mM Y27632 (Tocris) was added to PROL-T medium until complete organoids were generated. Culture medium of both normal and tumor organoids was changed every two days.

For passaging, we followed the protocol previously described [29] with some modifications. Briefly, organoids were incubated with 1 mg/mL of dispase (Thermo Fisher Scientific) for 30 min at 37 °C. Matrigel-embedded organoids were collected and 269 × *g* centrifuged for 5 min. Organoid pellets were disaggregated in 1:4 TrypLE Express (Gibco, NY, USA) PBS with 6 mM Y27632 for 5 min at 37 °C by passing through a 21 G syringe. After two washes in washing buffer, the pellets were embedded in Matrigel and seeded on culture dishes. Y27632 was present in PROL and PROL-T media until fragmented organoids or single cells generated complete organoids.

### Induction of cell differentiation

Colon normal organoids were cultured in PROL medium for 5 days and colon tumor organoids were cultured in PROL-T medium for 3 days before changed to the indicated differentiation medium: DIFF (PROL medium lacking L-WRN conditioned medium, RSPO1, Nicotinamide, SB-202190 and PGE_2_); BMP4 (DIFF medium lacking Noggin and supplemented with 20 ng/mL BMP4 [Gibco]); DBZ (DIFF medium supplemented with 2.5 µM DBZ [Tocris] in normal organoids and 5 μM in tumor organoids) and B + D (DIFF medium lacking Noggin and containing BMP4 and DBZ at the indicated doses). Culture media were changed every two days. Calcitriol effects were assessed by treating organoids with 100 nM calcitriol (Sigma-Aldrich) or vehicle (ethanol) in each condition. Light microscopy images were captured with a DFC550 digital camera (Leica, Wetzlar, Germany) mounted on an inverted TS100 microscope (Nikon, Tokio, Japan).

### Cell viability assay

Colon normal organoids (passages 8–13) were disaggregated in 1:4 TrypLE Express to single cell and 48,000 cells were seeded on 30 µl Matrigel (10 µl/drop) in 24-well culture dishes. Organoids were cultured in PROL medium plus Y27632 for 5 days. Then, organoids were washed twice in PBS and incubated in differentiation medium (DIFF, BMP4, DBZ or B + D) in the presence of 100 nM calcitriol or vehicle (ethanol). Medium and calcitriol/vehicle were replaced every other day. Upon 5 or 7 days of incubation of organoids in differentiation medium, cell viability was determined by estimating the amount of cellular ATP using the Cell Titer-Glo 3D Luminescent Cell Viability Assay (Promega) following the manufacturer’s instructions.

### Real-time quantitative PCR (RT-qPCR)

Matrigel-embedded normal (passages 4–12) and tumor (passages 23–64) organoids were washed twice in PBS and lysed with TRIZOL (Thermo Fisher Scientific). Total RNA was purified using the NucleoSpin miRNA extraction kit (Machery-Nagel, Düren, Germany), and for cDNA retrotranscription iScript cDNA Synthesis kit (Bio-Rad, CA, USA) was used. RT-qPCR analyses were performed with the Taqman® Universal PCR Master Mix (Applied Biosystems, CA, USA) using the following FAM-labeled TaqMan probes (Applied Biosystems): *LGR5* (Hs00173664_m1), *SMOC2* (Hs00405777_m1), *KRT20* (Hs003000643_m1), *CA1* (Hs01100176_m1), *KLF4* (Hs00358836_m1), *FABP2* (Hs00164552_m1), *AGR2* (Hs00180702_m1), *TFF2* (Hs00193719_m1), *TFF3* (Hs00902278_m1), *DKK1* (Hs00183740_m1), *ID2* (Hs04187239), *CYP24A1* (Hs00167999_m1). RNA expression values were normalized *vs*. the housekeeping genes *GAPDH* (Hs99999905_m1; FAM-labeled TaqMan probe) or *RPLP0* (H99999902_m1; VIC-labeled TaqMan probe) using the comparative C_T_ method. We also used Power SYBR® Green PCR Master Mix (Applied Biosystems) and the following primers to *VDR* (forward 5′-AACGCTGTGTGGACATCGGC-3′; reverse 5′-GTCATGGCTTTCGTTGGACT-3′) and *HES1* (forward 5′-TGAAGAAAGATAGCTCGCGG-3′; reverse 5′- GCGCAGCCGTCATCT-3′) detection. RNA expression values were normalized *vs*. the housekeeping gene *SDHA* (forward 5′- GGGAACAAGAGGGCATCTG-3′; reverse 5′- CCACCACTGCATCAAATTCATG-3′). All RT-qPCR were performed in a CFX384 Touch Real-Time PCR Detection System (Bio-Rad). Human SW480-ADH colon carcinoma cells were used to obtain a relative value to compare *VDR* expression (Supplementary Fig. S4).

### RNA-sequencing (RNA-seq)

Colon normal organoids from six patients (passages 2–9) were seeded in 12-well culture dishes with PROL medium. Five days later, they were incubated in B + D medium in the presence of 100 nM calcitriol or vehicle for 48 h. Total RNA samples (500 ng; RQS/RIN average 8.6, range 7.3–9.6) were converted into cDNA sequencing libraries with the “QuantSeq 3′ mRNA-Seq Library Prep Kit (FWD) for Illumina” (Lexogen, Cat.No. 015). Briefly, library generation is initiated by reverse transcription with oligodT priming, followed by a random-primed second strand synthesis. The resulting purified cDNA library was applied to an Illumina flow cell for cluster generation and sequenced on an Ilumina NextSeq 550 by following manufacturer’s protocols. Analyses were performed using Nextpresso RNA-seq pipeline [54]. Briefly, sequencing read quality and cross-contamination analysis were performed by FastQC and FastqScreen softwares, respectively. Raw reads were preprocessed using BBDuk software in order to trim polyA and trueseq adapters contaminant sequences and aligned to hg19 human genome assembly using Bowtie and TopHat aligners. Gene counts were generated from alignment maps using htseq-count function. Differential expression analysis was performed as paired test using DeSEQ2 R package. Gene Set Enrichment Analysis (GSEA) and single sample GSEA (ssGSEA) were carried out from logFC-ranked lists to assess the degree of association between our RNA-seq profile, and the different samples that comprises it, with other signatures defined with genes enriched (differentiation gene sets: brush border and cellular hormone metabolic process from MSigDB, goblet cells [55, 56] proximal and distal enterocytes [56]; proliferation gene sets: E2F targets and G_2_-M checkpoint from MSigDB; stemness gene sets: *EPHB2*^+^ cells [57], *LGR5*^+^ cells and *PTK7*^+^ cells [58]). Functional enrichment analyses were performed on differentially expressed genes with *g*:Profiler tool.

### Western blot

Normal organoids were routinely collected after removing Matrigel by dispase incubation, except in the phospho-SMAD assays in which Matrigel was not eliminated. Pelleted or Matrigel-embedded organoids were lysed using RIPA buffer followed by sonication (0.05 M Tris-HCl pH 7.5, 0.1% SDS, 0.15 M NaCl, 1% Triton X-100 [Sigma-Aldrich] and 1% sodium deoxycholate) containing protease and phosphatase inhibitors (10 μg/mL leupeptin, 10 μg/mL aprotinin, 1 mM PMSF, 1 mM orthovanadate, 25 mM β-glycerolphosphate and 1 mM NaF, all from Sigma-Aldrich). Whole-cell extracts (15–30 µg or 80 µg the case of phospho-SMAD analyses) were separated by SDS-PAGE, transferred to PVDF membranes and incubated with the following primary antibodies: rabbit polyclonal-CA1 (CUSABIO, TX, USA, #CSBPA004364GA01HU), mouse monoclonal-GAPDH (Abcam, Cambridge, UK, #ab8245), rabbit monoclonal-PTK7 (Cell Signalling, MA, USA, #25618), rabbit polyclonal-SMAD1/5/8 (Santa Cruz, TX, USA, #sc6031-R), rabbit monclonal-Phospho-SMAD1/5/8 (Cell Signalling, #9511 S) and rabbit polyclonal-TFF2 (Proteintech, IL, USA, #13681-1-AP). ImageJ was used for the semi-quantification of protein levels.

### Immunofluorescence and confocal microscopy

Normal organoids embedded in Matrigel were fixed in 3.7% PFA for 30 min at RT. Once they were collected in 1.5 mL tubes and centrifuged at 4500 × *g* for 3 min at 4 °C, they were postfixed again in the same buffer for 30 min and pellets were washed three times in PBS. Organoids were incubated for 30 min at RT in PBS containing 1% BSA and 0.1 M glycine to quench residual PFA and washed again. Organoids were then placed on microscope slides (Superfrost Plus, Thermo Fisher Scientific) in a drop of PBS, covered with coverslips, and dry ice frozen during 5 min. Coverslips were removed and slides were washed twice in PBS at 4 °C. Organoids were then permeabilized in PBS 0.5% Triton X-100 during 30 min at RT and washed four times in PBS 0.01% Tween-20. Non-specific sites were blocked by incubation in PBS 2% BSA at RT for 1 h. Organoids were then incubated overnight at 4 °C with the primary antibodies diluted in PBS 1% BSA: rabbit monoclonal-AGR2 (1/100, Cell Signalling, #13062), mouse monoclonal-E-cadherin (1/100, BD Biosciences, NJ, USA, #610182), rabbit polyclonal-KLF4 (1/500, Gene Tex, CA, USA, #GTX101509), mouse monoclonal-KRT19 (1/10, Progen, Germany, #61010), rabbit polyclonal-TFF2 (1/100, Proteintech, #13681-1-AP). After four washes, organoids were incubated with secondary antibodies conjugated with AlexaFluor-488 or −546 dye (1/250, Thermo Fisher Scientific) for 45 min at RT and washed. For F-actin staining, organoids were incubated with Phalloidin conjugated with AlexaFluor-546 (1/40, Thermo Fisher Scientific, #A22283) at RT for 1 h. For nuclear counterstaining, slides were incubated with DAPI (1/250, Thermo Fisher Scientific, #D1306) for 30 min at RT. Coverslips were mounted onto microscope slides with ProLong Diamond antifade reagent (Thermo Fisher Scientific). Confocal microscopy was performed with a Zeiss LSM510 laser scanning microscope using a 40X objective. For multiple labeling experiments, images of the same confocal plane were sequentially acquired using Zeiss Confocal software (Zen 2.3 SP1) by sequential excitation at 405/488/561 nm to detect DAPI, AlexaFluor-488 and AlexaFluor-546, respectively. Quantification was done using ImageJ software (National Institutes of Health, USA) by calculating the mean gray value (MGV) of a transverse plane of each organoid. MGV determines the gray values of the pixels divided by the number of pixels in a selected area (intensity/area), which is an average of fluorescence intensity within the selection.

### Electron microscopy

For ultrastructural analysis, samples from normal (passages 8–10) and tumor (passages 25–60) PDO cultures were fixed with 3% glutaraldehyde (Merck-Millipore, MA, USA) in 0.12 M phosphate buffer, pH 7.4 during 30 min. Later, they were collected in 1.5 mL Eppendorf tubes and centrifugated 4500 × *g* for 5 min 4 °C. Upon 2 h postfixation with the same fixative solution and centrifugation at 15,700 × *g* samples were washed three times in PBS for 15 min before storage at 4 °C. Samples were then rinsed in 0.12 M phosphate buffer, postfixed in 2% osmium tetroxide (Sigma-Aldrich), dehydrated in acetone, and embedded in ACM Durcupan (Fluka Sigma-Aldrich) at 60 °C for 2 days. Ultrathin sections (50–60 nm) stained with 2% uranyl acetate and lead citrate were examined with a JEM 1011 (JEOL, Japan) electron microscope, operating at 80 kV. Micrographs were taken with a digital camera (Orius 1200 A; Gatan, USA) and processed using Adobe Photoshop (24.0.0) (Adobe Systems). Semithin sections (1 µm thick) stained with toluidine blue were used for the light microscopy observation of the samples.

We considered two ultrastructural features for the determination of the differentiation grade of enterocytes: i) rootlet length and ii) microvilli density; and of goblet cells: i) number of clusters of secretory vesicles per cell, and ii) total cluster area per cell. These features were then assigned with arbitrarian values (1, 2 or 3) in order to define the cell differentiation grade as the sum of these values. According to this criteria shown in Supplementary Table S1, we classified enterocyte and goblet cell differentiation into three categories: low (L), medium (M) and high (H). This morphometric and quantitative analysis was performed on electron micrographs of longitudinally sectioned luminal cells of colonic organoids at a magnification of x8000 and conducted using ImageJ software. The quantification was performed blindly and agreed by two authors (AM and PB-M).

### Statistical analysis

Statistical analysis was performed using GraphPad Prism (GraphPad). Two-tailed Student´s *t*-test was used for pairwise and two independent groups comparison. Multiple comparisons were performed with ANOVA while Chi-square test was performed in contingence tables corresponding to cell countings in ultrastructural images. Finally, calcitriol effect on differentiation markers (Fig. 5B, D) was analyzed by One-sample *t*-test. *P < 0.05, **P < 0.01, and ***P < 0.001 were considered significant. Box plots represent median ± max/min. Graph bars represent mean ± standard error of the mean (SEM).

### Supplementary information

Supplementary Figures

Supplementary Table S1

Supplementary Table S2

Supplementary Table S3

Supplementary Table S4

Supplementary Table S5

Original WB

## Data Availability

The RNA-seq data were submitted to the Gene Expression Omnibus (GEO) under the accession code GSE248274. The full and uncropped western blot were uploaded as the Supplemental Material. Other datasets are available from the corresponding authors on reasonable request.
