# Supplementary material for: Vitamin D opposes multilineage cell differentiation induced by Notch inhibition and BMP4 pathway activation in human colon organoids
Source: Cell Death Dis. 2024 Apr 29;15(4):301. doi: 10.1038/s41419-024-06680-z (PMC11058856; doi:10.1038/s41419-024-06680-z)

SUPPLEMENTARY FIGURES LEGENDS

Supplementary Fig. S1 Representative images of colon healthy tissue of colorectal cancer patient #101. A Light microscopy image of an isolated crypt. **B-D** Ultrastructural images of tissue crypt middle/top area showing mature enterocytes (B), a goblet cell (C) and an enteroendocrine cell (D). **E-H** Ultrastructural images of the crypt bottom area, showing a panoramic view of the crypt (E), stem cells (F), a partially differentiated enteroendocrine cell (*, G), and a putative DCSC (**, H). Subepithelial fibroblasts (Fb) are indicated in panels (E) and (G).

Supplementary Fig. S2 Human colon normal organoids respond to BMP4 and DBZ. A Western blot analysis of phospho(P)-SMAD1/5/8 and total SMAD1/5/8 (patient #47) in normal organoids cultured in DIFF medium and treated with BMP4 in the presence or absence of Noggin for 1 h. **B** RT-qPCR analysis of the RNA levels of the BMP4 target genes *ID2* and *DKK1* following 8 h treatment in the same conditions as in A (patients #92, #110 and #159; mean ± SEM). **C** RT-qPCR analysis of the RNA level of the Notch target gene *HES1* following 8 h treatment with DBZ in DIFF medium (patients #92, #110 and #159; mean ± standard error of the mean (SEM), *$P < 0.05$).

Supplementary Fig. S3 Epithelial differentiation features of human colon normal organoids cultured in B + D medium. Low (upper panels) and high (lower panels) magnification images showing either pure enterocytic, pure mucosecretory, or mixed cell phenotype organoids.

Supplementary Fig. S4 Human colon normal organoids respond to calcitriol treatment. A RT-qPCR analysis of basal *VDR* RNA levels in organoids cultured in PROL medium. Values

are related to those found in human SW480-ADH colon carcinoma cells (mean ± standard deviation, SD); **B** *CYP24A1* induction by calcitriol for 48 h (patients #47, #86, #110, #130) or 72 h (patients #47, #110, #130, #159).

Supplementary Fig. S5 Calcitriol extends cell viability in human normal organoids under differentiation conditions.

A Light microscopy images of normal organoids (patient #110) cultured in PROL, DIFF, BMP4, DBZ or B + D media for 7 days in the presence of calcitriol (100 nM) or vehicle. **B** Cell viability of normal organoids from 3 patients (#47, #89, #110) cultured in the indicated media and treated with calcitriol (100 nM) or vehicle for 5 days (left panel) or 7 days (right panel) (mean ± SEM).

SUPPLEMENTARY TABLES

Supplementary Table S1. Criteria used to estimate the differentiation grade of enterocytes and goblet cells.

Supplementary Table S2. Number of cells displaying low (L), medium (M) or high (H) grade of differentiation in normal organoids incubated in DIFF, BMP4, DBZ or B + D media in the presence or absence of calcitriol (100 nM) for 48 h.

Supplementary Table S3. List of genes differentially regulated in colon normal organoids upon 48 h incubation in B + D medium identified by RNA-seq assay ($q < 0.05$). Patients #92, #158, #159, #161, #166 and #172.

Supplementary Table S4. List of genes regulated by calcitriol (100 nM) in colon normal organoids incubated in B + D medium for 48 h identified by RNA-seq assay ($q < 0.05$). Patients #92, #158, #159, #161, #166 and #172.

Supplementary Table S5. Clinico-pathological characteristics of the patients whose biopsies were used in this study to generate organoid cultures and mutational status of tumor organoids.

Supplementary Figure S1

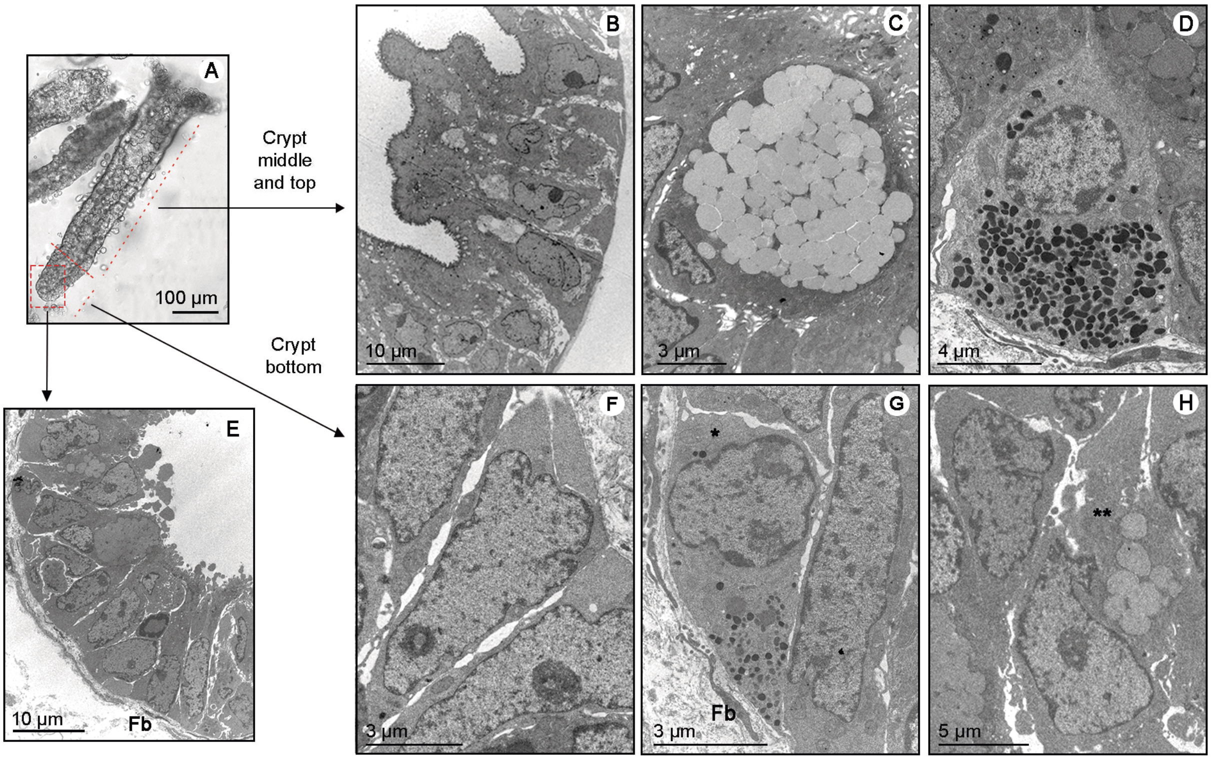

Supplementary Figure S2

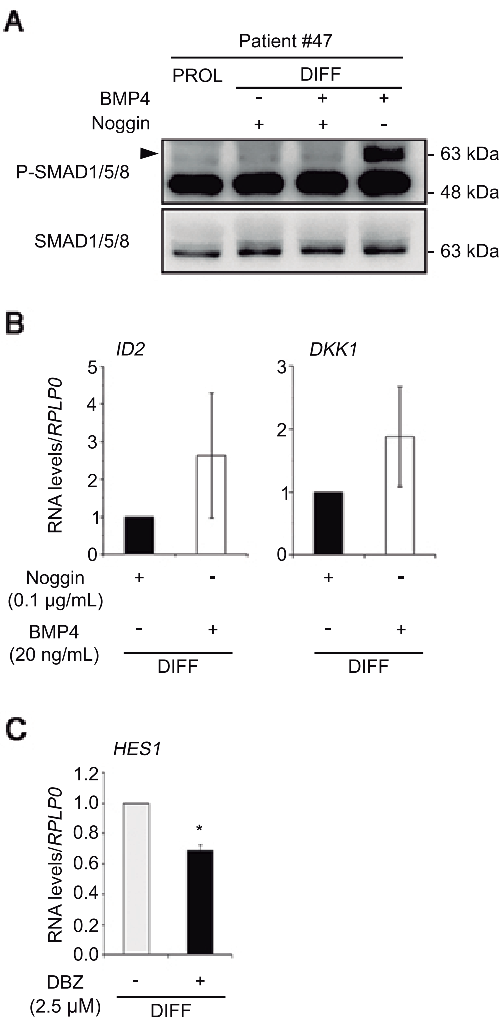

Supplementary Figure S3

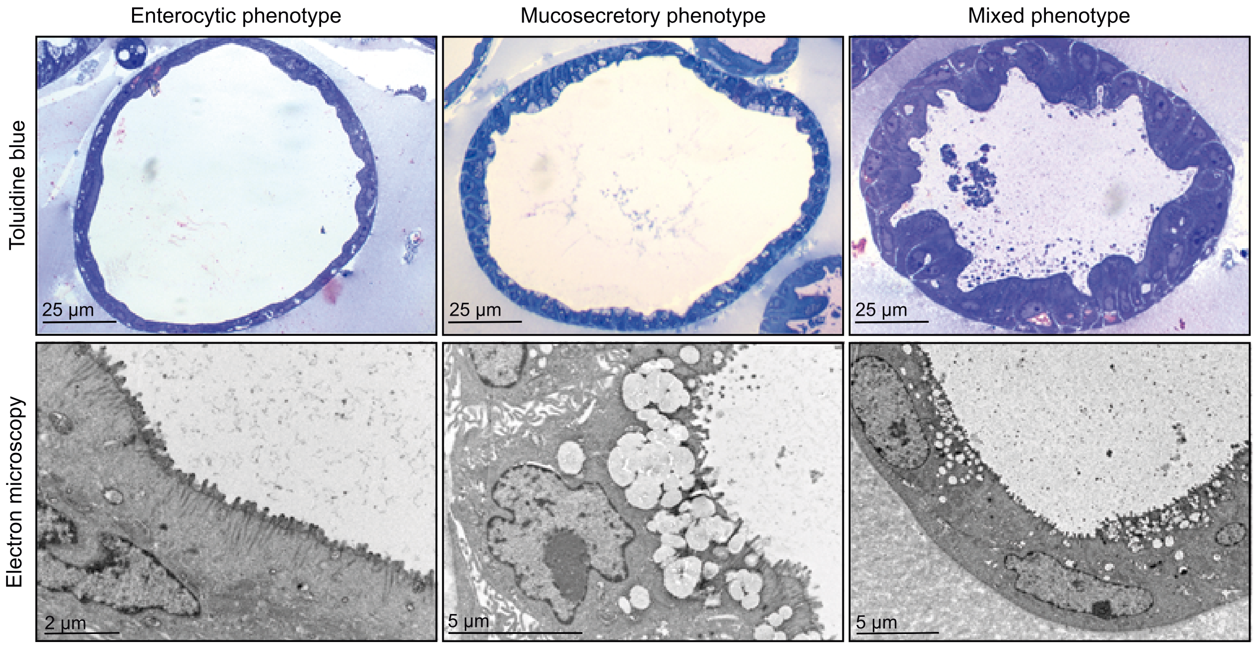

Supplementary Figure S4

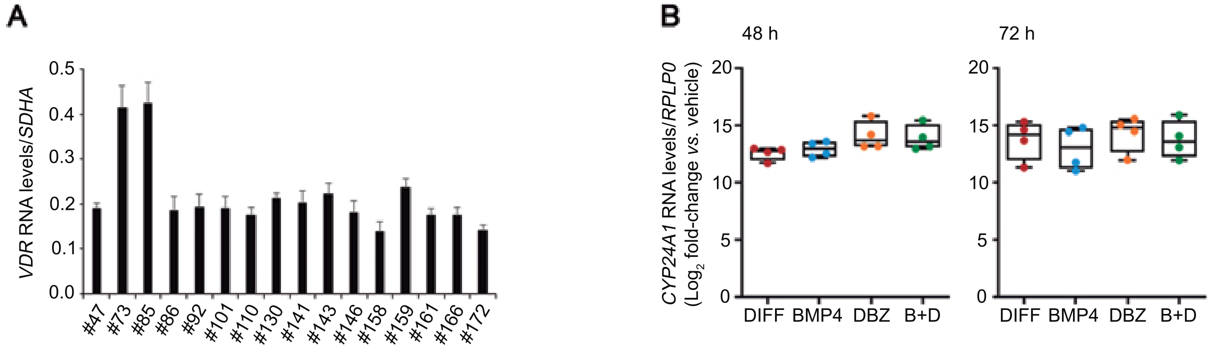

Supplementary Figure S5

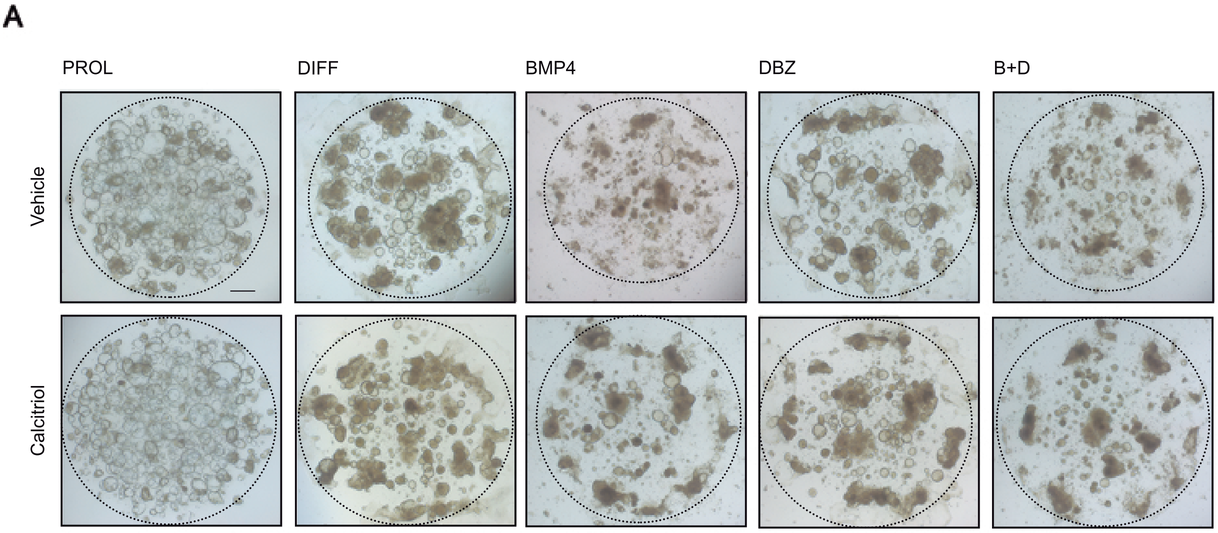

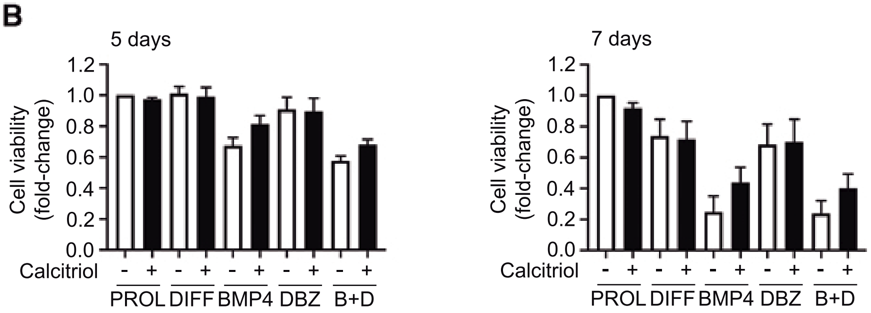

Supplement: Supplementary file 1 — Supplementary Figures [file 41419_2024_6680_MOESM1_ESM.pdf]
