# Supplementary material for: Vitamin D opposes multilineage cell differentiation induced by Notch inhibition and BMP4 pathway activation in human colon organoids
Source: Cell Death Dis. 2024 Apr 29;15(4):301. doi: 10.1038/s41419-024-06680-z (PMC11058856; doi:10.1038/s41419-024-06680-z)

# Original WB: Figure 5D.1

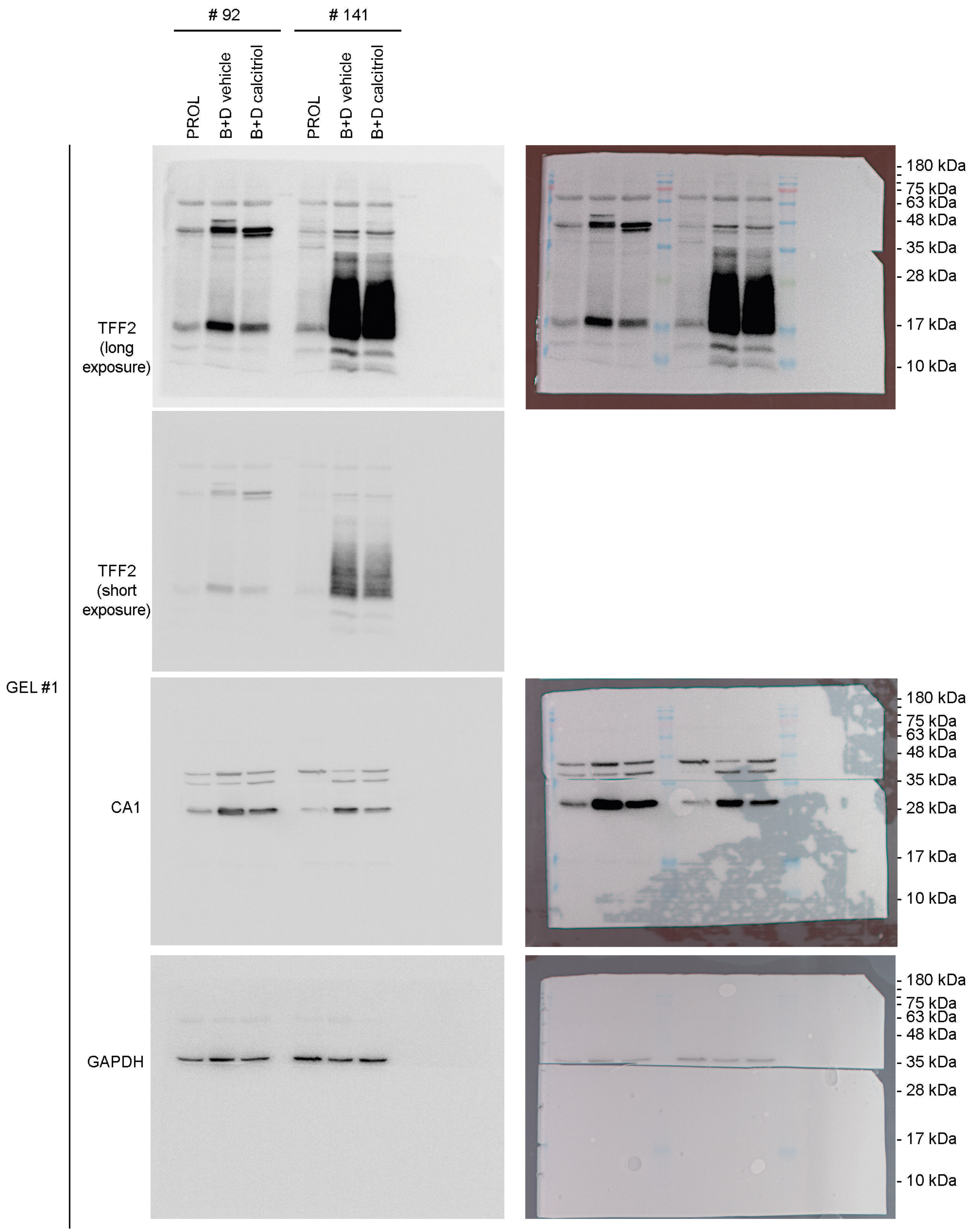


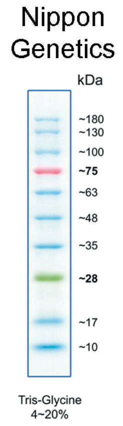

Original WB: Figure 5D.2

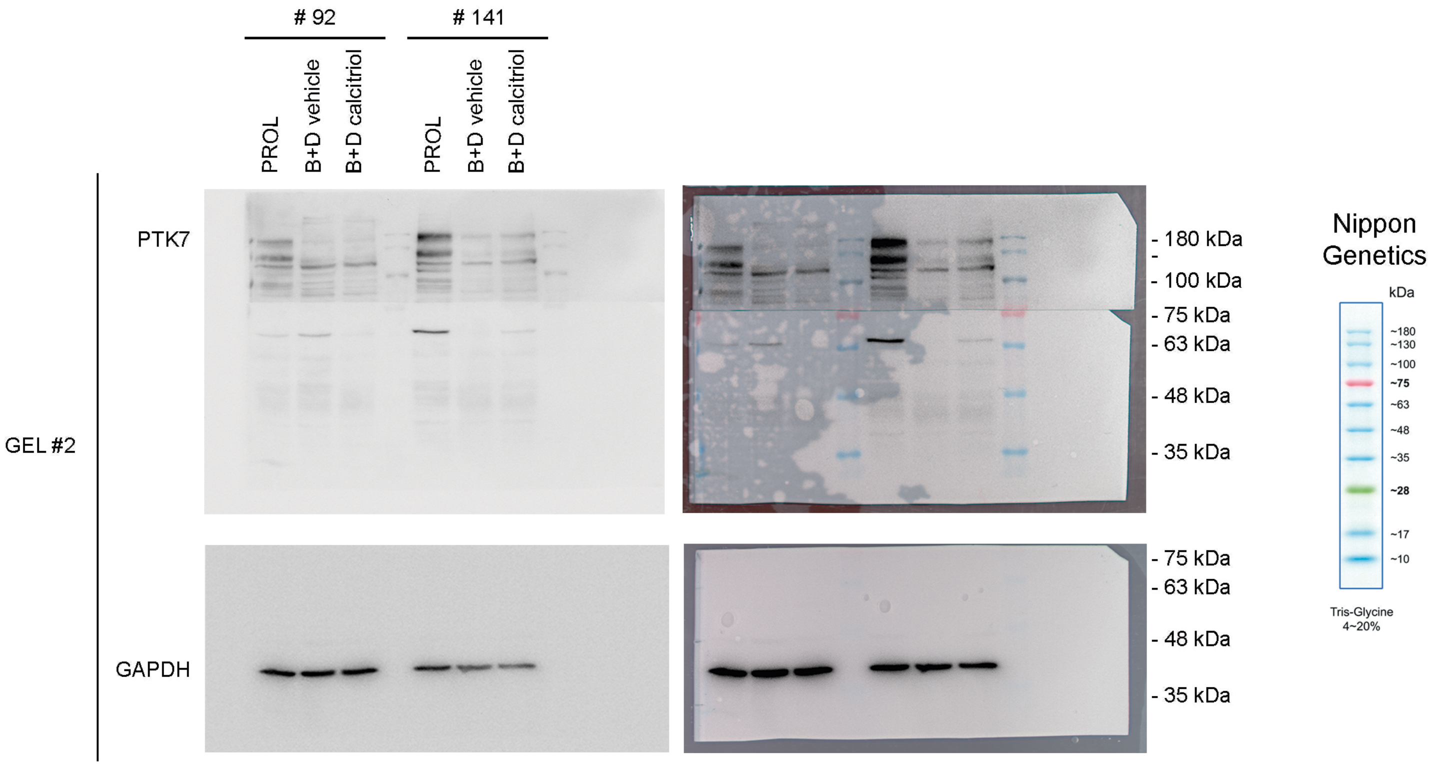

Original WB: Figure 5D.3

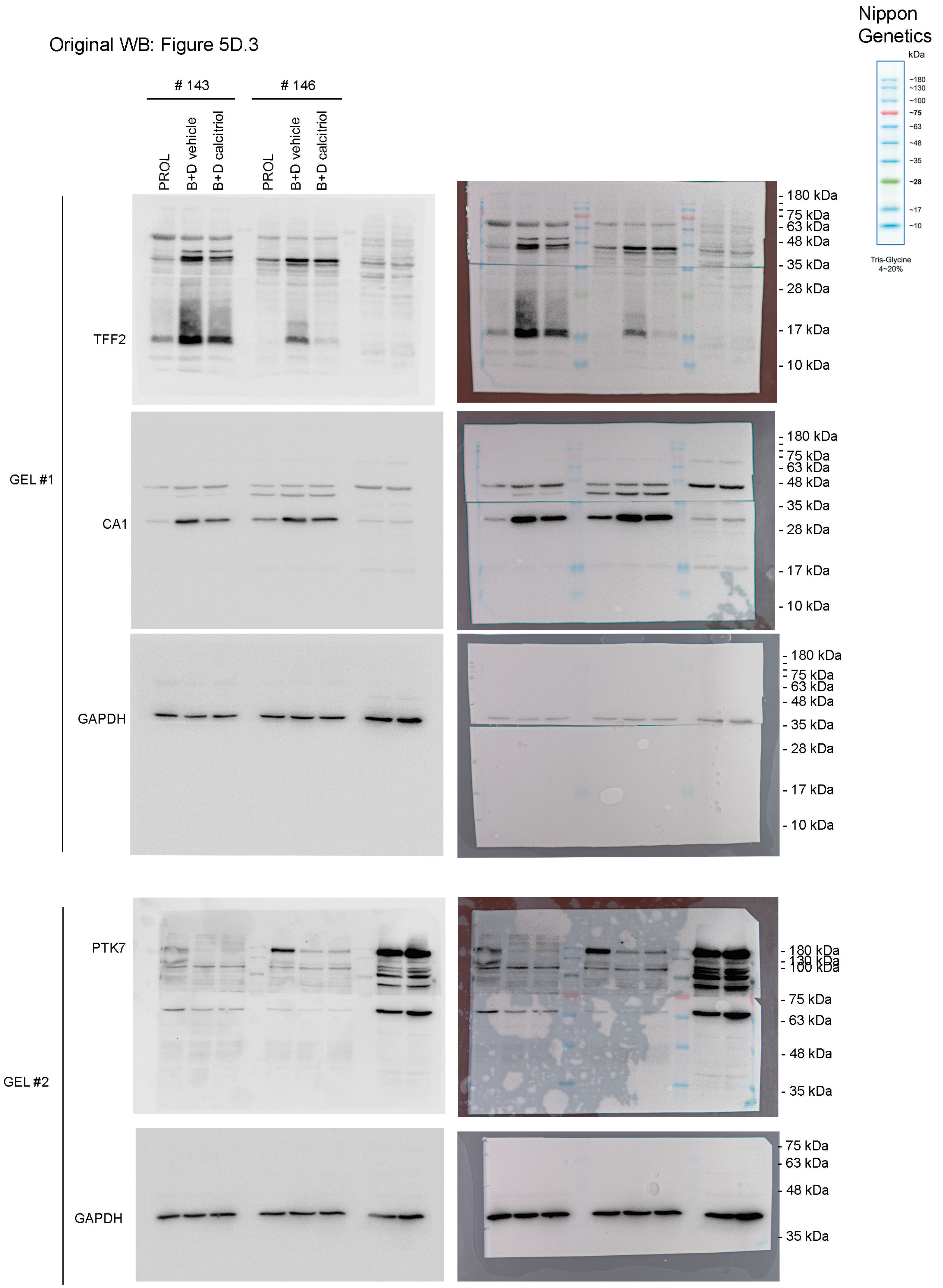

Original WB: Figure 5F

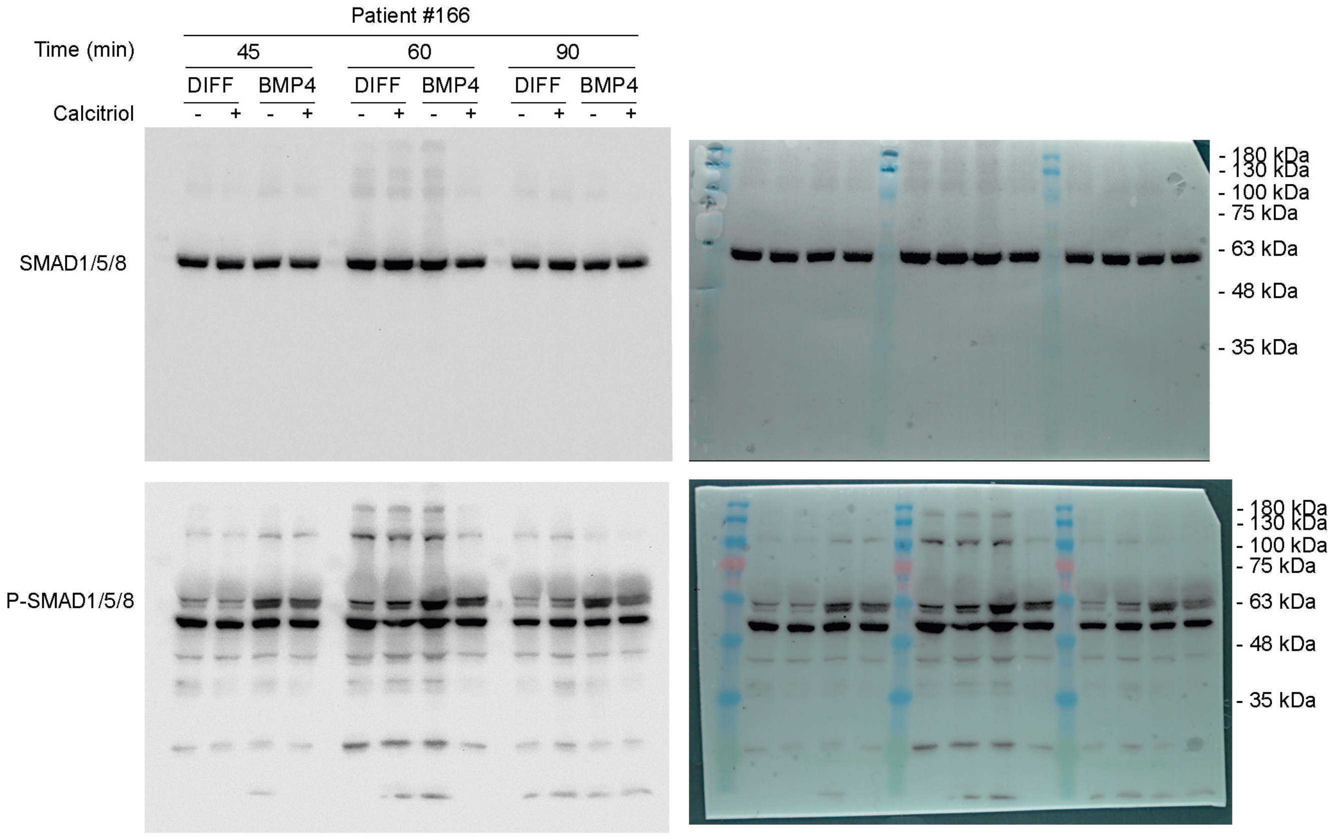


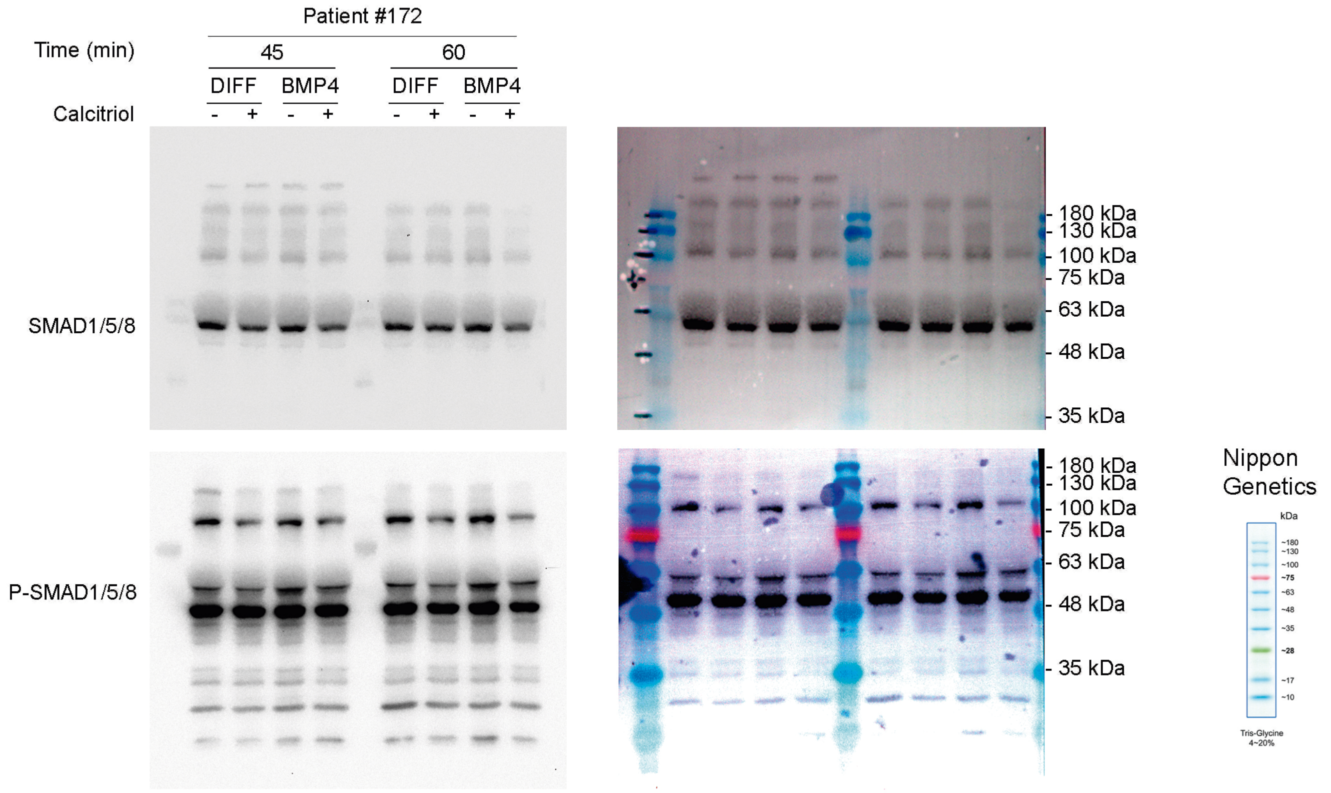

# Original WB: Supplementary Figure S2A

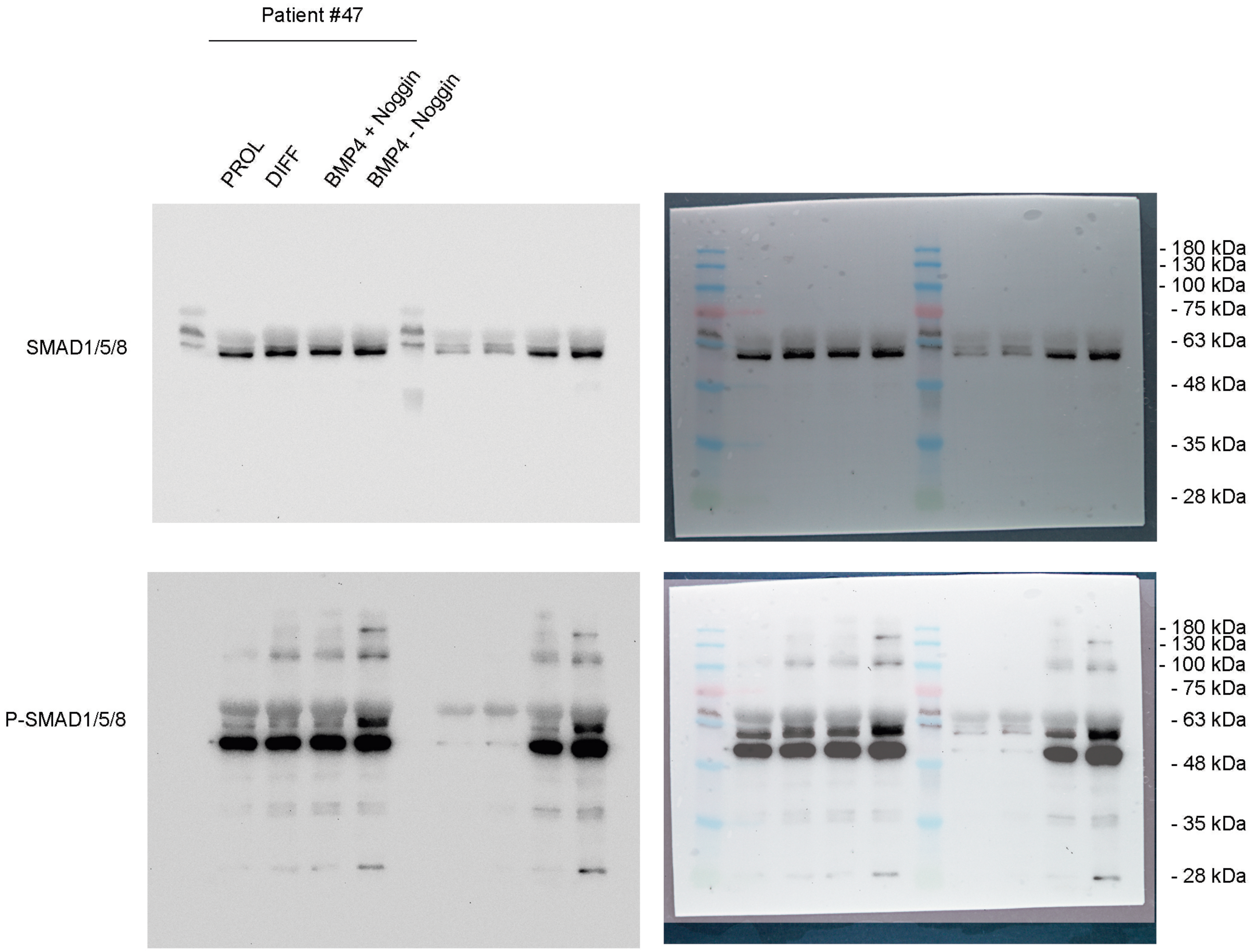


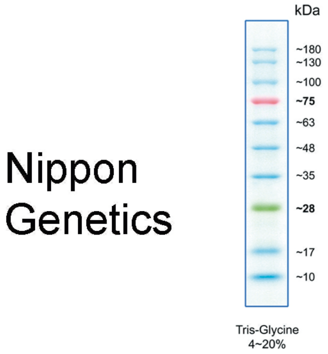

Supplement: Supplementary file 7 — Original WB [file 41419_2024_6680_MOESM7_ESM.pdf]
